# Supplementary material for: Tailoring digital apps to support active ageing in a low income community
Source: PLoS One. 2020 Dec 10;15(12):e0242192. doi: 10.1371/journal.pone.0242192 (PMC7728168; doi:10.1371/journal.pone.0242192)
Supplement: S1 File — (PDF) [file pone.0242192.s001.pdf]

Focus Group Script

HOUR 1:

1. Opening. Paula Castro or Grace Gomes (5 mins)

Explain the PAUL project and the co-design methodology
- Welcome
- Active and healthy aging
- Motivation for physical activities in an urban environment
- Monitoring and training apps
- Focus group method and re-design

2. Motivational video. Lua Bonadio (2 mins)

Video in which Drauzio Varella talks about the importance in his life of starting to run at the age of 50 in the urban environment of the city of Sao Paulo

3. Questions Part 1. Paula Castro (30 mins)

1. What do you think about having a trainer to help in performing the physical exercise?

2. Do you think motivational messages would help your practice? Because?

> *Maybe specify motivational messages. For example, when do user receive the message? Do they receive them during running? Do the messages serve as a reminder/suggestion/feedback or 'just' praise? Would they like to receive textual or audio messages?*

3. Some people like to play against themselves and improve their own performance, some like to play competitively against others and some like to play in a team. Which kind of person are you? Can you describe the way you like to play and measure your performance now?

> *I like this question! Maybe you can add if they would like to share their achievements/activities on social media.*

Other questions which could be relevant concern also more specific persuasive strategies such as:

1. Do you think that an activity goal will motivate you to perform more? What would be the requirements of a good activity goal? Would you prefer a self-set goal, or an automatic (tailored) goal?

2. Some questions on rewards? Virtual vs. real life rewards, quantity, etc.?

4. Pacer demonstration. Lua Bonadio (7 mins)

For the workshop will be available tablets with Android device for handling participants. The basic functionality of a Tablet will be presented initially, so that the members can use the tool with the maximum of autonomy. For those who are struggling the researchers present will offer help and support for the use and tasks.

After this process of (re) knowing the device, the next step is to present the application chosen as the basis for the co-design, "Pacer pedometer and coach". We will teach the basic functionality of the application, it is also intended to guide them to perform specific tasks of the application, such as choosing a personal goal to achieve within the listing available in the application.

To support this learning, we build presentations for display in the Datashow, showing the step-by-step of each task, guiding the right usability and intended by this project. These presentations will detail each topic of the tasks within the Tablet and within the Pacer application.

Questions: What do you think of this? Would you use it? Would it be uesful? Why?

[If there is time…6. An application for physical activity would be effective to stimulate the practice? 7. Would you like the active participation of your app data? 8. Would you like to talk to others who also do activities with you within the application?]

Cofee (15 mins)

<u>Hour 2:</u>

5. Re-design. (Paulinha, Lorena and Andresa moderators). (40 mins)

- Keep, Loose, Change -

This is when the participants will give their opinion about the application. After the content seen, the tasks performed, the understanding of the functionality and the digital trainer, the volunteers will be divide in the digital engagement subgroup and

1. Perform the tasks using Pacer
2. Point out what they liked in the application to be maintained, what they did not like to be removed and finally the new aspects to be added, some suggestion topic in the current application that could make a difference in a new one to be developed.

These suggestions will be collected by drawings and texts on cardstock, where participants will be able to draw and write a new design, also make comments on topics,

ideas for tools. We will also collect data through recording, identifying shared oral ideas beyond what is on the physical paper.

6. Show New Designs To Each Other (15 Mins)

- Come back together. One spokesperson or the facilitator to show designs of each group to the other group for feedback.
