# Supplementary material for: Tailoring digital apps to support active ageing in a low income community
Source: PLoS One. 2020 Dec 10;15(12):e0242192. doi: 10.1371/journal.pone.0242192 (PMC7728168; doi:10.1371/journal.pone.0242192)
Supplement: S2 File — (PDF) [file pone.0242192.s002.pdf]

Roteiro do Grupo Focal

Hora 1:

1. Abertura. Paula Castro ou Grace Gomes (5 mins)

Explicar o projeto PAUL e a metodologia Co-design
- Recepção
- Envelhecimento Ativo e Saudável
- Motivação para atividades físicas em ambiente urbano
- Aplicativos de monitoramento e treinamento
- Método de grupo focal e re-design

2. Vídeo Motivacional. Lua Bonadio (2 mins)

Vídeo no qual Drauzio Varella fala sobre a importância em sua vida de começar a correr aos 50 anos no meio urbano da cidade de São Paulo.

3. Questões Parte 1. Paula Castro (30 mins)

1. O que você acha de ter um treinador para ajudar na realização do exercício físico?

2. Você acha que mensagens motivacionais ajudariam na sua prática? Porque?

> *Talvez especifique mensagens motivacionais. Por exemplo, quando o usuário recebe a mensagem? Eles os recebem durante a corrida? As mensagens servem como lembrete / sugestão / feedback ou "apenas" elogio? Eles gostariam de receber mensagens textuais ou de áudio?*

3. Algumas pessoas gostam de jogar contra si mesmas e melhorar seu desempenho, algumas gostam de jogar competitivamente contra outras pessoas e outras gostam de jogar em equipe. Que tipo de pessoa você é? Você pode descrever como gosta de jogar e medir seu desempenho agora?

> *Talvez você possa adicionar se eles gostariam de compartilhar suas realizações / atividades nas redes sociais.*

Outras questões que podem ser relevantes dizem respeito também a estratégias persuasivas mais específicas, tais como:

1. Você acha que uma meta de atividade o motivará a realizar mais? Quais seriam os requisitos de uma boa meta de atividade? Você prefere uma meta definida por você mesmo ou uma meta automática (personalizada)?

2. Algumas perguntas sobre recompensas? Recompensas virtuais vs. reais, quantidade, etc.?

4. Demonstração do Pacer. Lua Bonadio (7 mins)

Para o workshop estarão disponíveis tablets com dispositivo Android para atendimento aos participantes. As funcionalidades básicas de um Tablet serão apresentadas inicialmente, para que os membros possam utilizar a ferramenta com o máximo de autonomia. Para aqueles que estão com dificuldades, os pesquisadores presentes oferecerão ajuda e suporte para o uso e as tarefas.

Após este processo de (re) conhecimento do dispositivo, o próximo passo é apresentar a aplicação escolhida como base para o co-design, "Pedômetro e treinador de marcapasso". Ensinaremos as funcionalidades básicas do aplicativo, também se destina a orientá-los para a realização de tarefas específicas do aplicativo, como escolher um objetivo pessoal a atingir dentro da listagem disponível no aplicativo.

Para apoiar esse aprendizado, construímos apresentações para exibição no Datashow, mostrando o passo a passo de cada tarefa, orientando a usabilidade correta e pretendida por este projeto. Essas apresentações detalharão cada tópico das tarefas no Tablet e no aplicativo Pacer.

Perguntas: O que você acha disso? Você usaria? Seria útil? Por quê?

[Se houver tempo… 6. Um aplicativo de atividade física seria eficaz para estimular a prática? 7. Você gostaria da participação ativa dos dados do seu aplicativo? 8. Você gostaria de conversar com outras pessoas que também realizam atividades com você no aplicativo?]

Cofee (15 mins)

<u>Hora 2:</u>

5. Re-design. (Paulinha, Lorena and Andresa moderadoras). (40 mins)

- Keep, Loose and Change -

É nesse momento que os participantes darão sua opinião sobre o aplicativo. Após o conteúdo visualizado, as tarefas realizadas, a compreensão da funcionalidade e do treinador digital, os voluntários serão divididos no subgrupo de engajamento digital e

1. Executarão as tarefas usando o Pacer

2. Indicarão o que gostaram no aplicativo e que deve ser mantido, o que não gostaram e que deve ser retirado e por último os novos aspectos a serem adicionados, algum tópico de sugestão no aplicativo atual que pode fazer a diferença em um novo a ser desenvolvido.

Essas sugestões serão coletadas por meio de desenhos e textos em cartolina, onde os participantes poderão desenhar e escrever um novo design, também fazer comentários sobre temas, ideias de ferramentas. Também coletaremos dados por meio de registros, identificando ideias orais compartilhadas além do que está no papel físico.

6. Mostrar novos designs uns aos outros (15 Mins)

- Se reunir. Um porta-voz ou facilitador deve mostrar os designs de cada grupo ao outro grupo para feedback.
