# Supplementary material for: Tailoring digital apps to support active ageing in a low income community
Source: PLoS One. 2020 Dec 10;15(12):e0242192. doi: 10.1371/journal.pone.0242192 (PMC7728168; doi:10.1371/journal.pone.0242192)
Supplement: S1 Questionnaire — (PDF) [file pone.0242192.s003.pdf]

Pre-interview questionnaire

In this document, we present the questionnaire developed by the authors and used for the pre-interview of the participants.

There were scales other than those presented in this document that were applied to the same subjects because this study was part of a larger project (Playful Datadriven Active Urban Living), but these data were not used in the present study.

A. THE IDENTIFICATION

A.1 Name:____________________________________ Telephone: (   )______________

A.2 Age:__________Date of Birth:____/_____/______ A.3 Gender:____________

A.4 Education (years):__________

A.5 Retired: (1) Yes (0) No

A.6 Occupation:____

B. TOPICS OF THE OF THE INTERNATIONAL PHYSICAL ACTIVITY QUESTIONNAIRE (IPAQ)

C. SMARTPHONE DATA

C.1 Do you have a cell phone?

(1) Yes (If the answer to question 1 is positive, skip to question C.3)

(0) No (If the answer is no, go to next question C.2)

C.2 If your answer was negative, why don't you have a cell phone? (Go to section F)

____

____

C.3Do you have a smartphone with a touchscreen?

(1) Yes (If yes, go to section D)

(0) No

C.4 Why don't you use a smartphone with a touchscreen?

(1) Difficulty in use

(2) Demotivation

(3) I don't think it's necessary

(99) Don't know/didn't answer

(4) Another reason. Which one? ___________________________________

C.5 What is the brand/model of your cell phone?

C.6 What is your smartphone's operating system?

(1) IOS-Iphone (2) Android (3) Windows Phone

D. HOW SMARTPHONE IS USED

D.1 How often do you use your smartphone?

(4) Every day

(3) Several days a week

(2) Once or twice a week

(1) Rarely, one to three times a month.

D.2 Where do you use your smartphone most often?

(1) Public places - streets, squares, public transportation, etc.

(2) Private places like stores, banks, supermarkets, etc.

(3) At home

(4) Anywhere, I don't care about it

(5) Other. Which one?__________________

D.3 What tasks do you perform most on your smartphone? (You can choose as many tasks as you want)

D.3.1 Answering and making calls (1) Yes (0) No

D.3.2 Alarm (1) Yes (0) No

D.3.3 Whatsapp, Facebook, Twitter or other networks that involve interaction (1) Yes (0) No

D.3.4 Health-oriented management applications (1) Yes (0) No

D.3.5 Viewing and writing emails (1) Yes (0) No

D.3.6 GPS or traffic navigation applications (1) Yes (0) No

D.3.7 Contacts (1) Yes (0) No

D.3.8 Activity management (1) Yes (0) No

D.3.9 Games (1) Yes (0) No

D.3.10 News (1) Yes (0) No

D.3.11 Weather / climate (1) Yes (0) No

D.3.12 Others. Which are?_____________________________________

D.4 Do you take your smartphone during your training / physical activity section?

(3) Yes, always

(2) Yes, sometimes

(1) Rarely

(0) I don't take it.

D.4.1 If not, why not? ______________________________________

E. HOW APPS ARE USED

E.1 What applications have you used today?

____

E.2 Are you currently using an application aimed at physical activity or health? Which one?

____

E.3 Do you usually download free or paid physical activity or health apps?

____

E.4 How do you know the applications you download?

____

E.5 Which functions of your application related to physical activity or health do you use most?

____

F. BEHAVIORAL REGULATION IN EXERCISE QUESTIONNAIRE – BREQ 3 - BRAZILIAN VERSION
