# Supplementary material for: Tailoring digital apps to support active ageing in a low income community
Source: PLoS One. 2020 Dec 10;15(12):e0242192. doi: 10.1371/journal.pone.0242192 (PMC7728168; doi:10.1371/journal.pone.0242192)
Supplement: S2 Questionnaire — (PDF) [file pone.0242192.s004.pdf]

Questionário da Pré-entrevista

Neste documento, apresentamos o questionário desenvolvido pelos autores e utilizado para a pré-entrevista dos participantes.

Havia escalas diferentes das apresentadas neste documento que foram aplicadas aos mesmos sujeitos porque este estudo fazia parte de um projeto maior (Playful Datadriven Active Urban Living), mas esses dados não foram usados na presente pesquisa.

A. IDENTIFICAÇÃO

A.1 Nome:__________________________________ Telefone: (   )______________

A.2 Idade:______Data de Nascimento:____/_____/______ A.3 Gênero:___________

A.4 Escolaridade (anos):__________

A.5 Aposentado: (1) Sim (0) Não

A.6 Ocupação:____

B. TÓPICOS DO INTERNATIONAL PHYSICAL ACTIVITY QUESTIONNAIRE (IPAQ)

C. DADOS DO SMARTPHONE

C.1 Você tem aparelho celular?

(1) Sim (Caso a resposta da questão 1 seja positiva pule para a questão C.3)

(0) Não (Caso a resposta seja negativa vá para a próxima questão C.2)

C.2 Se sua resposta foi negativa, por que você não tem aparelho celular? (Vá para seção G)

____

C.3Você possui smartphone com tela sensível ao toque (touchscreen)?

(1) Sim (Caso a resposta seja positiva, vá para a seção D)

(0) Não

C.4 Por que você não utiliza smartphone com tela sensível ao toque?

(1) Dificuldade de utilização

(3) Não acho necessário

(2) Desmotivação

(99) Não sabe/não respondeu

(4) Outro motivo. Qual? ____

C.5 Qual a marca/modelo do seu aparelho celular?

C.6 Qual o sistema operacional do seu smartphone

(1) IOS-Iphone (2)Android (3)Windows Phone

D. COMO SE DÁ O USO DO SMARTPHONE

D.1 Com qual regularidade você utiliza seu smartphone?

(4) Todo dia

(3)Vários dias por semana

(2) Uma ou duas vezes por semana

(1) Raramente, uma a três vezes por mês.

D.2 Onde você utiliza seu smartphone com mais frequência?

(1) Locais públicos - ruas, praças, transporte coletivo, etc.
(2) Locais privados como lojas, bancos, supermercados, etc.
(3) Em casa
(4) Em qualquer lugar, não me importo com isso
(5) Outro. Qual?__________________

D.3 Quais tarefas você mais executa em seu smartphone? (Você pode escolher quantas tarefas quiser)

D.3.1 Atender e efetuar chamadas (1) Sim (0) Não

D.3.2 Alarme (1) Sim (0) Não

D.3.3 Whatsapp, Facebook, Twitter ou outras redes que envolvem interação (1) Sim (0) Não

D.3.4 Aplicativos de gerenciamento voltado à saúde (1) Sim (0) Não

D.3.5 Visualizar e escrever e-mails (1) Sim (0) Não

D.3.6 GPS ou aplicativos de navegação de trânsito (1) Sim (0) Não

D.3.7 Agenda de contatos (1) Sim (0) Não

D.3.8 Gerenciamento de atividade (1) Sim (0) Não

D.3.9 Jogos (1) Sim (0) Não

D.3.10 Notícias (1) Sim (0) Não

D.3.11 Tempo/clima(1) Sim (0) Não

D.3.12 Outros. Quais?____________________________________

D.4 Você leva seu smartphone durante sua seção de treinamento / prática de atividade física?

(3) Sim, sempre
(2) Sim, às vezes
(1) Raramente
(0) Não levo.

D.4.1 Se não leva, por quê? _____________________________________

E. COMO SE DÁ O USO DOS APPS

E.1 Que aplicativos você já utilizou hoje?

____

E.2 Hoje você está utilizando algum aplicativo voltado à atividade física ou saúde? Qual?

____

E.3 Você costuma baixar aplicativos de atividade física ou saúde gratuitos ou pagos?

____

E.4 Como você conhece os aplicativos que baixa?

____

E.5 Quais as funções do seu aplicativo voltado à atividade física ou saúde você mais utiliza?

____

<u>F. BEHAVIORAL REGULATION IN EXERCISE QUESTIONNAIRE – BREQ 3 – VERSÃO BRASILEIRA</u>
