# Supplementary material for: Tailoring digital apps to support active ageing in a low income community
Source: PLoS One. 2020 Dec 10;15(12):e0242192. doi: 10.1371/journal.pone.0242192 (PMC7728168; doi:10.1371/journal.pone.0242192)
Supplement: S1 Transcript — (PDF) [file pone.0242192.s005.pdf]

ÁUDIO SUFICIENTEMENTE ATIVOS:

Pesquisador: Então começando agora de verdade, né, se acalmando, boa tarde a todos, nós queríamos agradecer demais a presença de vocês hoje. A professora Pesquisador trabalha bastante nesta área do envelhecimento ativo e de como se manter ativo ao longo de toda a vida, fazendo exercícios, atividades físicas, e agora ela ta com um projeto novo, quero dizer, novo dentro daquilo que ela já estuda, que é como fazer isso dentro da cidade que a gente mora, o bairro que a gente mora, então em vez de ir a um lugar específico pra fazer exercício, como uma academia por exemplo, a gente usar a cidade para isto, ir até a igreja, usar a rua pra fazer uma caminhada, etc e tal pra fazer atividade física. Então hoje nós vamos conversar com vocês como é isso pra vocês, fazer atividade física ao longo da vida e pra isso nós vamos mostrar uma pessoa que começou a fazer atividade física que tinha mais de 50 anos, que é o doutor Celebridade Brasileira, vocês devem conhecer! Conhecem? Ele é bem popular! O Doutor Celebridade Brasileira começou a correr ele tinha mais de 50 anos porque ele foi no médico, outro médico amigo dele, e ele tava prestes a ter um problema de coração, e por isso ele precisava começar a fazer atividade física. Ele nunca tinha feito e começou com mais de 50 anos e hoje ele corre na cidade de São Paulo, em maratona, ele corre bastante. Então ele vai falar um pouco dessa experiência e depois eu vou fazer umas perguntas pra vocês sobre como vocês acham que é fazer exercícios, o que motiva vocês, e depois vocês vão ajudar a gente dando ideias pra como seria se fossemos desenvolver um programa de celular pra ajudar a pessoa a praticar atividade física na cidade, como se fosse um treinador no celular, tá? Então é isso Pesquisador, quer acrescentar alguma coisa?

Pesquisador: Quero também agradecer todo mundo que veio, estamos super felizes, muito obrigada. Estou muito feliz de rever algumas pessoas do grupo da caminhada né? Espero que vocês também estejam caminhando bastante, fazendo atividades físicas, e hoje é um dia super importante pra gente porque a gente quer ouvir a opinião de vocês, porque nós estamos tentando montar esse programa no celular para oferecer as pessoas aqui do bairro, então precisa ser uma coisa do bairro, uma característica daqui, com o que vocês pensam, então por isso temos que ouvir a opinião de vocês. Adianta ouvir a opinião de quem tá na universidade? Não adianta, tem que ser vocês, vocês que vão dar opinião "ah acho que isso aqui tá certo, ou, acho que não tá certo não", dizer pra gente a opinião, dar ideias de como funcionaria um bom programa de celular. Depois a professora Pesquisador e as alunas vão entrar em detalhes, explicar como isso aconteceria, mas a ideia geral é que vocês deem a opinião. Então por isso nós vamos falar sobre atividade física e na sequencia falar sobre as oponiões que vocês tem "ah eu acho legal, funcionaria, ou então pra minha vizinha daria certo mas pra outra pessoa não daria certo" tá? Então é isso, queremos a opinião de vocês. Não da certo a gente perguntar para as pessoas de fora do bairro. Por que? Porque eles não conhecem, são vocês que vivem aqui, não é? Então por isso é muito importante ouvir de vocês aqui pra gente. Ta bom? Então agradeço muito, eu vou precisar sair aqui um pouquinho, no meio da nossa atividade, porque eu tenho um curso que estou fazendo la na UFSCAR, mas vocês vão ficar com a professora Pesquisador e com as alunas e espero que vocês aproveitem bastante essa atividade, e depois tem o café da tarde antes de ir embora. Ta bom? Queria agradecer novamente vocês terem vindo. Agora a professora Pesquisador vai passar o vídeo.

(Vídeo Motivacional do Dr. Celebridade Brasileira)

Pesquisador: Então, tava um pouco baixo, né, então vou contar pra vocês o que ele falou, tá? É.. A gente ouviu na minha sala e tava tão alto, vocês me desculpem. Ele conta que até os 50 anos ele viveu uma vida muito pouco saudável, tanto de alimentação, quanto atividade física, ai ele tomou essa decisão né, pra proteger o coração dele, de começar a correr, e que ele é apaixonado pelo centro da cidade de São Paulo, aqueles prédios antigos, tem muita arvore, ele gosta de correr no meio das arvores, no meio das pessoas e das ruas, né, e que isso mudou a vida dele, ele não seria a pessoa que ele é hoje se não tivesse começado a correr, se ele não fosse corredor. E aí ele deixa o exemplo dele, o que que isso mudou na vida dele, né? É.. e aí eu queria ver, vou fazer algumas perguntas pra vocês a respeito disso, vocês são todos ativos né, vocês que estão aqui todos fazem atividade física. Alguns aqui no grupo, alguns caminham né, aqui pelo bairro perto, caminhada, exatamente. Vocês acham que ajuda ter orientação, ou seja, alguém que vai falar pra vocês como fazer o exercício, mesmo se fosse no celular, ou quando vocês vem aqui na aula tem um professor que fala pra vocês?

Participante: Sim!

Participante: Sim, ajuda bastante..

Participante: Sims

Participante: Eu vi passar um senhor de 90 e tantos anos, acho que 97, que caminha todos os dias. Ele trabalhava na roça sabe, de sol a sol, ficou forte fazendo essas atividades, a roça as vezes é o exercicio que tem acesso

Pesquisador: Exatamente, quem morava na fazenda costuma ser mais ativo, fazer mais exercício, né

Participante: É verdade

Participante: Eu mesma morava na fazenda, então trabalhava na roça, fazia muito trabalho, nunca parava

Pesquisador: Nunca ficou parada né?

Participante: É, tinha sempre muito trabalho pra fazer, já trabalhei demais

Pesquisador: Entendo.. Como é o nome da senhora?

Participante: é Participante.

Pesquisador: Dna Participante, então na sua opinião, depois que a senhora mudou da fazenda para a cidade foi mais difícil fazer atividade física? Fica mais parada?

Participante: Fica mais parada, fica mais parada sim

Pesquisador: Porque a senhora acha que a cidade não ajuda?

Participante: a cidade não tinha exercício pra fazer onde eu morava não tinha um lugar, não tinha logo que eu vim pra cá, né, mas aí eu não saía direito, antes eu tava acostumada a fazer caminhada, sair e voltar pra minha casa, ia na casa da minha mãe certinho, mas num tem lugar

Pesquisador: Então na comunidade que a senhora morava antes não tinha espaço? Não tinha campinho, espaço?

Participante: Nada! Não tinha nada. Já na roça tinha lugar, tinha trabalho, tinha campinho, um riozinho perto da praia,sempre tava pra lá e pra ca, nos campos perto do rio

Pesquisador: E o rio? A senhora acha que é importante ter o rio? É bom quando tem uma água?

Participante: Sim, era muito bom

Participante: É lá em casa não dava, agora não tem mais 35 anos e não tem

Participante: É verdade, quando eu comecei a morar aqui na aracy...

*sussurros baixos*

Pesquisador: Quer falar?

Participante: Não precisa não

Pesquisador: Pode falar! A gente quer ouvir a opinião de todo mundo!

Participante: Não, não...

Participante: Pode falar! Pode falar

Pesquisador: O que que é? É sobre o espaço?

Participante: Não, porque, é assim, se desse pra fazer um espaço de ginástica pra nós seria bem melhor

Pesquisador: Entendi

Participante: Como o centro da juventude lá, não tem mais, e tá precisando, viu!

Participante: Eu ia toda semana praticar

Participante: É verdade, lá a professora fazia junto, tinha zumba também

Participante: Verdade, a gente precisava disso daí

Pesquisador: Então salas? E piscina? O Que vocês acham? E lugar, assim, amplo por exemplo pista de caminhada, marcar as ruas assim, os metros, se colocasse assim, os metros nas ruas e marcar

Participante: Se tivesse uma pracinha!

Pesquisador: Pracinha?

Participante: Pra fazer as atividades seria bom. Porque pra gente aqui pegar ônibus é difícil, tem lugares que oferecem atividades mas é longe, precisava ser no centro da juventude perto da gente

Pesquisador: Onde? No Campo do ruy? Kartódromo?

Participante: Não é ali na, subindo a Getulio Vargas

Pesquisador: Aahh sei, lá perto do Centro de Especialidades. No CRI né?

Participante: Sim, o Centro de Especialidades

Participante: Sim, o CRI. É! Lá mesmo. Você já foi la?

Participante: Agora esse ano acho que não fui nenhuma vez

Pesquisador: O que tem de bom lá que falta aqui? De espaço?

Participante: Ah, tem as salas né, tem um espaço bom para as pessoas, a moça ajuda a gente na academia,

Participante: Tem a escola né. Tem um espaço lá para as pessoas

Participante: Tem a academia pra gente ir lá fazer

Pesquisador: Tem alguém que supervisiona a atividade lá?

Participante: Tem sim, tem sim. E agora com esse negócio de ônibus a gente não sabe quando da pra ir, né. Por causa disso nem fui ainda

Pesquisador: Eu tenho outra pergunta pra vocês. Vocês falaram que ajudaria ter alguém orientando vocês, né, e ajudando e estimulando. Vocês acham que se esse alguém fosse pelo celular, como uma mensagem de celular, também ajudaria?

Pesquisador: Vocês gostariam de receber? Não, to falando assim, pra quem tem, se tivesse um celular, vamo imaginar que a pessoa tem um celular

(sussurros baixos)

Pesquisador: Não? Por que?

Participante: É a pessoa, porque o que ajuda é cara com cara

Pesquisador: Cara com cara! Então você gostaria que tivesse um rosto.. Entendi. E se no celular tivesse uma pessoa de verdade, como se ela tivessse te telefonando?

Participante: Seria bem melhor

Participante: No celular é bem melhor

Pesquisador: É melhor? Então vocês acham, ó, então vocês acham que essa mensagem seria boa, mas ela teria que ter um formato como se fosse uma pessoa te ligando, e ai ela teria que ter um rosto e uma voz?

Participante: É, mas precisa ter o telefone pra saber

Pesquisador: É

Participante: Aí da pra usar né, pelo celular

Pesquisador: Uhum

Participante: Agora pega o telefone e liga, vem aqui e resolve tudo

Participante: Verdade *risos*

Pesquisador: Entendi. E se fosse uma mensagem que deixasse no celular, como se fosse um vídeo, igual esse do doutor Dráuzio, mas curtinho, aí você aperta e ouve a hora que você quiser, não precisa atender. E aí essa pessoa ta falando pra você sobre o exercício

Participante: É como eu tava falando pra você, ajuda sim

Pesquisador: Quantas pessoas aqui tem celular? Levanta a mão

Pesquisador: Metade! Metade, metade

Participante: Eu tenho

Participante: Sim, o meu celular..

Participante: Aqui meu celular!

Participante: Eu não tenho!

Participante: Preciso aprender a mexer

Pesquisador: Nós temos uma oficina!

Pesquisador: Então vocês prefeririam ter alguém falando do que ler uma mensagem escrita? O que preferem? Uma mensagem por escrito ou uma mensagem com a voz de alguém falando?

Participante: Pra mim tanto faz!

Pesquisador: Tanto faz, entendi...

(sussurros baixos)

Pesquisador: Oi??

Participante: Eu não sei ler (risos)

Pesquisador: Então Pra você teria que ser alguém falando?

Participante: É, teria que ser

(riso geral)

Participante: E se fosse como era antigamente? Não pode mais?

Pesquisador: Como assim?

Participante: Porque antigamente a gente saía do postinho, né, e fazia as atividades todos juntos

Pesquisador: Ah, entendi. Você gostaria que tivesse uma pessoa junto

Participante: Sim, na caminhada né, atividade, ajuda

Pesquisador: Entendi. Você gostaria que tivesse todo o tempo da atividade tivesse alguém ajudando, não somente com uma mensagem

Participante: Sim

Pesquisador: Monitoramento, a pessoa vai te acompanhar de perto

Participante: Isso, isso

Pesquisador: E vai te acompanhar todo o tempo que você ta fazendo a atividade

Pesquisador: Todo mundo aqui tem neto? E aí, vocês já viram o neto de vocês usar o celular?

Participante: Sim, mexem sem parar noite e dia

Pesquisador: Vocês já observaram o uso de celular por eles, quem nunca teve contato com celular? Vocês já observaram como que é o celular?

Participante: É meu neto mexe sem parar

Pesquisador: Sem parar né

Participante: Sim, fica mexendo a noite

Participante: Eu já vi sim

Pesquisador: Vocês já ouviram que eles recebem algumas mensagens as vezes? As vezes tem coisa escrita, é como a Pesquisador estava falando, tem como apertar um botão pra ver a mensagem

Participante: Já vi sim, minha sobrinha mexe e eu já vi

Pesquisador: Tem a possibilidade de ouvir a mensagem, é isso que a professora estava falando, com a voz de outra pessoa

Participante: Um passa pro outro né. Isso que é uma coisa interessante, as vezes né, aí ele passa comunica

Pesquisador: Isso, isso!

Pesquisador: Eu tenho mais uma pergunta sobre essa coisa da pessoa e da mensagem. Vamos imaginar, tanto faz se é uma pessoa ou se é uma mensagem que vai ta recebendo. Vocês gostam dessa pessoa que vai te acompanhar? Ou essa mensagem? Que ela fale pra você o que você tem que fazer e quais são os seus objetivos, qual vai ser o exercício do dia ou vocês também gostam que essa pessoa anime vocês, como "você ta indo super bem, você já fez 'tanto' de atividade hoje", que elogia, vocês gostam que elogia?

Participante: Sim, anima bastante todo elogio, né

Participante: Aí fica feliz demais

Participante: Daí fica interessante

Participante: Sim eu gosto sim, sim

Pesquisador: É? Você acha que te anima? Quando uma pessoa fala, te elogia, o elogio te deixa inspirado a fazer mais exercício?

Participante: Sim, fico animada

Pesquisador: E música? Vocês gostam de música?

Participante: Eu adoro música!

Participante: Música anima o ritmo da, né, atividade, sim

(risos e murmúrios gerais)

Pesquisador: Ok. Agora nós vamos mudar. Agora vamos supor que vocês tão fazendo atividade física, mas tem outras pessoas fazendo, por exemplo, um grupo de caminhada, o grupo de quando vocês vem aqui na igreja, pra fazer a atividade. Vocês preferem competir, de jogar contra as outras pessoas, ou vocês gostam de fazer colaboração, por exemplo uma gincana, todo mundo ajuda todo mundo a ganhar, ou melhor, vocês gostam de fazer o de vocês mesmos, a outra pessoa que faça o dela e acabou?

Participante: Gosto quando um ajuda o outro

Pesquisador: Colaboração, todo mundo junto

Participante: Verdade, eu também

Pesquisador: É? Quem aqui gosta de competir pra ver quem é melhor?

(Silêncio Geral)

Pesquisador: Ninguém?

Participante: Ah, eu gosto sim

Participante: Eu gosto de competição!

Participante: Ah sim, eu sou o melhor aqui

Pesquisador: Você também gosta, entendi

Pesquisador: O que você gosta na competição? O que você acha legal? Motiva? Anima?

Participante: Quando compete. A pessoa ganha, fica na frente né

Participante: Ah, quando compete anima, né, *risos*

Participante: Verdade, da vontade de fazer mais

Pesquisador: Quer fazer mais?

Participante: É!

Pesquisador: Então tem uma parte que gosta de competição e a outra de colaboração? Entendi

Pesquisador: Qual o lado bom de colaborar? E qual o lado bom de competir?

Participante: Ah tem aquele que chega primeiro

Participante: Aí ganha né *risos*

*riso geral*

Pesquisador: Ok. E qual o lado bom de ser colaborativo? E de competir? Vamos supor, quando é colaborativo todo mundo tem que chegar junto no mesmo objetivo

Participante: Colaborar?

Pesquisador: Sim, colaborar. "Todo mundo aqui agora tem que chegar naquela porta lá, juntos" aí vocês vão levantar e todo mundo vai junto. Vai ter alguém ai que vai falar "não, agora eu vou rápido e chegar primeiro" daí isso é competir, né, então qual é a vantagem de competir e qual a vantagem de colaborar?

Participante: Colaborar é melhor

Participante: Geralmente na ginastica nas atividades o que a gente faz é competir, né

Pesquisador: Ok, ela acha que na ginastica e atividades físicas é melhor competir

Pesquisador: E vocês gostariam, tem alguém aqui que gosta de fazer atividade física só sozinho? Por exemplo gosta de correr, de fazer sem ninguém

Participante: Eu! Gosto de fazer sozinha. Por isso gosto de fazer atividade aqui, a gente faz e eu fico no meu canto lá, e é melhor

Pesquisador: Entendi. Mas você também gosta de ter seu tempo só pra você. Porque?

Participante: Gosto, minha caminhada eu faço sozinha

Participante: Você quer andar né, daí tem seu limite, outra pessoa tira a concentração do momento. Quando conversa já não pega aquele ritmo que precisa pra fazer. Aí tem uma pessoa do seu lado que não anda igual você, aí precisa esperar o ritmo do outro

Pesquisador: Quem aqui tem facebook, essas coisas? Tem alguém que tem facebook, ou o neto tem facebook, vocês já viram alguém usando essas coisas?

Participante: Meu neto tem sim

Participante: Eu tenho

Participante: Eu também

Pesquisador: Vocês gostariam de poder compartilhar o que vocês fizeram, por exemplo, que vocês conseguiram fazer uma aula de ginástica, zumba ou cumpriu todas as caminhadas que precisava naquela semana, de compartilhar isso com o neto de vocês, ou com as outras pessoas do grupo de caminhada

Participante: Da minha parte sim

Participante: Eu também!

Participante: Sim seria legal

Pesquisador: Certo, vocês gostariam que o celular de vocês comunicasse com o celular do neto, dos amigos?

Participante: Sim

Participante: Quando tem um celular que da pra fazer isso é bom, né, aí todo mundo vê o progresso

Participante: Eu gosto de fazer minha caminhada sozinha, porque aí cabe na calçada né, mais gente eu acho perigoso

Pesquisador: Vocês acham... Opa desculpa, vocês queriam falar alguma coisa? Pode falar

Participante: Não só tava conversando aqui com ela

Pesquisador: Ah sim

Participante: Nós tavamos falando que eu prefiro fazer minha caminhada sozinha, porque sozinha cabe na calçada, né

Pesquisador: Ah isso é importante, então a cidade não cabe todo mundo andando na calçada, e fica perigoso, vocês sentem medo né

Participante: Vou sozinha na caminhada, daí tem o salão né, todo mundo faz junto, mas na caminhada eu prefiro sozinha, saber andar

Participante: Eu fazia caminhada todo dia as 5 e meia da manhã, uma hora de caminhada, eu ia da minha casa até a pista, só que aí começou a acontecer umas coisas, sabe, uns homens que ficam atrás da mulherada aí, então eu parei porque fiquei com medo, porque ia só eu e mais uma. Mas eu acho assim, que fazer caminhada em duas ou três é bom, porque a gente vai conversando e vai também distraindo, desabafando, e pra mim foi muito bom. Mas eu parei por causa disso, e voltaria a praticar atividade sim, tenho vontade

Pesquisador: Ta. E vocês acham que se você tiver que cumprir um resultado final, isso ajuda vocês no exercício? Ou por exemplo, você fala, "ah eu vou caminhar, mas tenho que caminhar 1 hora", uma meta, ou não, vocês acham estressante? Como que é isso pra vocês? Vocês gostam de ter que cumprir uma meta, um objetivo ou não?

Participante: Eu gosto

Participante: Eu gosto

Participante: É, verdade

Participante: No meu caso sim eu gosto, ajuda bastante

Participante: É bom né, pra saúde da pessoa

Pesquisador: Vocês acham que ajuda então? Por exemplo, se não rua tivesse marcado, ou uma praça, se tivesse marcado os metros no chão, aí você pode falar "nossa já andei 1 quilômetro, agora vou andar 2" ajuda?

Participante: Ajuda

Participante: Ajuda bastante

Participante: Sim, eu gostaria

Participante: Tinha uma vizinha minha lá do CDHU, muita gente praticava lá, um pasto, corre, faz exercício naquele sol

Pesquisador: Assim, uma pessoa é diferente da outra pessoa, né, vocês acham assim que esse objetivo, preferem que tenha um objetivo que seja pra todo mundo, como esse que eu falei dos quilômetros marcados na rua ou vocês prefeririam que tivesse um objetivo pra você, que alguém fizesse um específico pra você, pessoal

Participante: Igual pra todas pessoas

Participante: É, pra todo mundo, eu acho

Pesquisador: Entendi, pra todo mundo

Participante: É ué, vai fazer um objetivo pra uma pessoa só?

Pesquisador: Não, assim, por exemplo, cada pessoa ter o seu individual, por exemplo se o senhor conseguiu andar 2 quilômetros, seu objetivo na semana que vem vai ser andar três, mas a outra pessoa que só consegue andar 1 quilômetro o objetivo dela vai ser andar um e meio, por exemplo, aí são diferentes. Vocês entenderam o que eu to tentando dizer?

Participante: Entendi. As vezes algumas pessoas na caminhada leva criança, aí tem que ficar olhando os outros e perde a concentração

Pesquisador: Verdade, então seria melhor se fosse individual mesmo. E o senhor, comoé o nome do senhor?

Participante:  Participante!

Pesquisador: Como?

Várias Pessoas: Participante!

Pesquisador: Ah, Participante! Fala meio alto que eu sou surda eu Participante

Pesquisador: Você disse que prefere fazer sozinho?

Participante: Sim, aí individual ninguém atrapalha

Pesquisador: Se fosse para o senhor ter um objetivo assim, que o senhor tivesse que cumprir e fosse individual seu, porque o senhor gosta mais de fazer sozinho. Mas assim, você gostaria que tivesse alguém te ajudando?

Participante: Não

Pesquisador: Não? Você acha que você sabe e consegue fazer sozinho?

Participante: Sim

Participante: Sim, sim. Sempre faço atividade, como servente de pedreiro, é exercicio pesado sabe, eu faço sempre

Pesquisador: Gente, e se fosse pra vocês receberem uma recompensa, um prêmio, por exemplo, você vai ganhar, vamos supor que você consiga fazer 2 horas de servente de pedreiro ou então

andar 1 quilômetro, aí alguém fala pra você "se você conseguir andar 2 quilômetros semana que vem você vai ganhar 100 reais"

Participante: Aí eu ia *risos*

Participante: Quem não ia ne?

Participante: Aí eu faria muitos quilômetros sim *risos*

Participante: Verdade! *risos*

(riso geral)

Participante: Como é seu nome fia?

Pesquisador: Pesquisador!

Participante: Meu marido vai fazer 80 anos e trabalha o dia inteiro como servente de pedreiro com a carriola

Pesquisador: Então ele faz exercício o dia inteiro?

Participante: Sim

Pesquisador: Aí ele ganha a recompensa no final do mês!

Participante: Meu trabalho é andar, sou vendedora caminho desde manhazinha, ou saio meio dia e chego quatro hora da tarde

Pesquisador: E se a recompensa fosse outra que não fosse dinheiro, por exemplo, uma foto de uma flor, ou então uma mensagem legal pra motivar, alguma coisa assim, ou pontos em um jogo, vocês acham que seria interessante também?

Participante: Ah, seria né

Participante: Seria sim

Pesquisador: E se fosse assim por exemplo, aí teria mais a ver com competição, mas aí você seria eleito o melhor daquele grupo porque você fez mais exercício aquela semana, também? Ou não? Vamos supor, tem o grupo que faz atividade aqui na igreja, toda semana elege qual é o melhor, ou não?

Participante: Aí não

Pesquisador: Seria estranho?

Participante: Eu gostaria sim

Pesquisador: Ela gostou alá! Já está até pondo a coroa *risos*

(riso geral)

Participante: Aí motivaria sim, né *risos*

Pesquisador: Aí quem faz atividade física, a recompensa da semana pra quem fez mais exercício, seria escolhido o melhor. Aí por exemplo, teria uma foto dessa pessoa

Participante: Interessante sim, sim

Pesquisador: O senhor gostaria? E se tivesse um troféu?

Participante: Sim, eu acho interessante

Participante: Eu acho um trofeu, qualquer presente que a gente ganhe, troféu, não importa o tamanho dele, o que importa é qualquer coisinha pra gente é de valor, nem que seja um lembrete. Motiva a gente, igual que eu falei, se a gente for fazer caminhada com esforço por exemplo, por tudo que eles fizeram aqui a atividade precisa ser valorizada, né. Aqui é um espaço bom, aqui tem um espaço grande, todo o povo vai ficar contente, por exemplo, horário, marca o horário, eu no meu caso esse horário é bom pra mim caminhar uma e meia, tres quatro hora já não da mais, minha esposa também, o horario tem a ver com a caminhada também. Vamos supor que daqui saia 15, 20 pessoas pra caminhar, teria qur ter uma pessoa responsável pra ajudar, um guarda municipal, não sei, porque no bairro não cabe todo mundo na calçada, fica perigoso muita gente, dificulta

Pesquisador: Então pro senhor poder escolher o horário é muito importante?

Participante: Sim, importante o horário

Participante: Verdade, eu gosto de caminhar a tarde mas tem que goste de caminhar de manhã, né

Participante: Deixa eu perguntar. Já ouvi o doutor falar que trabalhar não é a mesma coisa de fazer atividade física, tipo assim, se você ta fazendo faxina não é considerado atividade, é uma coisa que só vai prejudicar, é verdade?

Pesquisador: Como é o nome da senhora?

Participante: Participante!

Pesquisador: Participante fez uma pergunta muito importante. Ela perguntou assim, as vezes que a gente faz atividade física no formato do nosso trabalho, por exemplo limpar a casa, limpar o jardim, essas coisas, né, é assim, quando a gente faz atividade física, igual naquela entrevista que vocês responderam para as meninas, tem atividade física que a gente chama de leve, tem atividade física que a gente chama de mais ou menos, e tem atividade física que é muito forte, então assim, a atividade física que é considerada forte, ela precisaria que o coração acelerasse um pouco mais, que é igual a caminhada, ou as atividades que vocês fazem aqui na igreja. Essas atividades físicas que a gente faz trabalhando jardinagem, limpar a casa, servente de pedreiro não, servente de pedreiro é pesado, elas são leves, mas elas são atividades físicas sim, ta? Elas são atividades físicas sim

Participante: Eu ouvi que não é igual

Pesquisador: Pois é, quando a gente ta lavando roupa a gente fica na posição errada, então o que seria legal, por exemplo, se tivesse um treinador, aí eu to falando que pode ser uma pessoa

ou pode ser esse que estamos discutindo em formato digital, ele poderia ensinar como lavar roupa na posição certa, entendeu, pra não prejudicar as costas. E meu coração, como ele ta enquanto eu to lavando roupa? Entendeu? Isso que eu tava falando de objetivo pessoa, que você lava a roupa, não outra pessoa, daí indicaria um exercício só seu, entendeu, uma coisa individual

Participante: Ah! Entendi

Pesquisador: Gente, agora a Pesquisador vai mostrar pra vocês, essa é a Pesquisador

Pesquisador: Prazer gente!

Pesquisador: Ela vai mostrar pra vocês um programa de celular, a gente chama de aplicativo, que conta os passos que a pessoa da durante o dia, e aí ele faz umas outras coisas também, e ela vai mostrar pra vocês que coisas são essas

Participante: Como é o nome?

Pesquisador: É Pesquisador

Participante: Você não vai conseguir contar meus passos não! *risos*

Pesquisador: Não, nós não vamos contar os passos de ninguém, nós só vamos mostrar o que o aplicativo do celular faz pra vocês verem. Pode começar Pesquisador.

-----

GRUPO INSUFICIENTEMENTE ATIVOS:

Pesquisador: Risos. Então eu vou pô aqui o gravadorzinho. E ai o que que nós vamos fazer com os resultados? Boa pergunta. Então esses resultados vão ser mandados lá para a Holanda, junto com os resultados dos estudos que estão sendo feitos lá, que são iguais esses que a gente está fazendo aqui, para a gente desenvolver um aplicativo de celular, um produto de celular, que nos ajude a fazer atividade física na cidade, de alguma forma, né? E como as vezes esses ap, esses produtos, que saem para celular eles não são adaptados para a gente, para a nossa realidade, nós que moramos aqui na cidade de São Carlos, que temos uma idade de 40, 50 ou 60 ou 70 anos, que moramos no Aracy, etc e tal. Então a nossa ideia é desenvolver um produto que seja adaptado para vocês, é por isso que a opinião de vocês é que vai decidir o que é que vai ter nesse produto, né? E ai esse produto que vai ficar pronto daqui uns 2 anos, vai ser lançado ai então no mercado e vocês vão poder usar obviamente, gratuitamente e ai a professora Pesquisador vai ligar para vocês quando estiver pronto. Participar talvez de uma segunda fase, né, de convidar vocês para participar de uma segunda fase que é testar o produto antes de ele sair do mercado, mas o mais importante é isso: Por que que as vezes é muito difícil usar um produto de celular? Ou de internet? Porque ele é feito de pessoas muito jovens para pessoas muito jovens, então as vezes a gente não tem nem interesse de usar, né? É. então esse é que é o problema e esse produto se é para fazer atividade física ao longo da vida então ele tem que sair de acordo com a opinião de vocês, né?

Participante: Porque tem muita gente que nem sabe usar, né? As pessoas mais velhas.

Pesquisador: É, exatamente.

Pesquisador: Então, a primeira coisa que nós vamos ver aqui é um vídeo do Dr. Celebridade Brasileira, vocês conhecem o Dr. Celebridade Brasileira? que fala né? É. Ele começou a correr aos 50 anos de idade, antes ele não fazia atividade física nenhuma, ai ele começou a correr aos 50 anos de idade e ele corre nas ruas de São Paulo. Então vamos colocar um vídeo dele falando como que é a experiência dele de correr.

(Vídeo do Dr. Celebridade Brasileira)

Pesquisador: Oh, então agora eu vou contar para vocês o que que ele falou. Risos.

Participante: É, infelizmente o áudio...

Pesquisador: Ele fala que quando ele fez 50 e poucos anos de idade ele não tinha vivido uma vida muito saudável, né? e ai ele tem alguns problemas de saúde e ele decide então começar a correr para melhorar a saúde dele e ai ele se apaixona por correr e ele gosta muito de correr na cidade de São Paulo, principalmente naquela parte do centro velho, tanto porque tem os prédios mais velhos quanto porque tem muita árvore e ele gosta de correr no meio das árvores. E ele fala que isso mudou a vida dele, que ele não seria quem ele é hoje se ele não tivesse começado a correr, né? É... E ele corre realmente essa proposta né, de o que a gente estava falando aqui, de correr na cidade, de fazer o seu exercício no bairro, na cidade, que é o que ele fala de centro velho, das árvores. E ai eu tenho umas, umas questões que eu queria que vocês me desse a opinião de vocês, assim. Vocês acham que para fazer uma atividade física assim, é, ou na cidade ou aqui na igreja ou em qualquer lugar, seria interessante ter alguém ou algum treinador que ajudasse vocês, que desse orientações?

Participante: Para incentivar.

Pesquisador: Incentivar também.

Participante: Até mesmo ligar antes de vir.

Pesquisador: Ligar? Você gostaria que...

Participante: Se a moça não tivesse para mim ontem, é, estou falando com a experiência da minha pessoa.

Pesquisador: Uhum.

Participante: Eu, eu tô em serviço, eu trabalho com coisa perecível, eu não podia tá aqui, quando você falou de 2 horas eu fiquei assim. Larguei minha filha olhando o carro, vendendo milho, que eu vendo milho ali, para vim até aqui. Mas como ela fez um pé de ir até minha casa, depois de um ano depois que a gente falou com ela e depois ela me ligou ainda: “Oh, vai ter, tal”, eu falei eu vou, eu vou fazer esse sacrifício de deixar minha filha lá olhando, ela não está bem de saúde, eu falei vou subir até lá para participar dessa reunião sim, porque eu acho que eu, várias vezes que eu comecei o grupo de caminhada por falta de incentivo.

Pesquisador: Essa coisa de ligar, de receber uma ligação ou de receber uma mensagem de incentivo então você achou legal?

Participante: Eu acho. Eu acho, porque a comunicação é muito boa como missão cumprida, para ver um sorriso, um “vamo lá” sabe “vai ser muito bom para sua saúde”, eu estou péssima de saúde, eu cheguei passando mal aqui, eu vim dali passando mal aqui, eu vim passando mal, agora melhorei, sentei, respirei e eu melhorei, sabe? Mas eu estou nesses elementos eu já estou com duas guias de fisioterapia e ainda não consegui fazer, uma que não tem tempo, toda vez que você vai é aquela burocracia, sabe? de pôr na vaga, de procurar isso, de procurar aquilo, então eu acho que devia de ser coisas assim mais fácil da gente fazer, entendeu?

Participante: Eu acho, assim...

Pesquisador: Ai desculpa.

Participante: que aqui, aqui nesse centro de juventude ter atividades todos os dias para a gente fazer, eu já estou com 4 anos que eu participo aqui, então a gente tem professor, se a gente tem 2 professores ai tem quem vai mais rápido, quem vai mais lento, eu faço caminhada, tudo, mas sempre tem alguém....Vai da 19 anos que eu participo desse estudo e para minha saúde foi muito bom.

Participante: Então, esse item que ela está falando de ter aquela pessoa que acompanha aquela que vai mais rápido e aquela pessoa que acompanha os que vai mais devagar, então quando eu comecei a fazer caminhada eu desisti por causa disso, toda vez minha pressão subia e eu passava mal, ai o grupo ia embora e largava eu para trás.

Pesquisador: Se fosse para fazer sozinha era melhor?

Participante: Quer dizer, se eu caísse e desse um infarto em mim ali não tinha nem um monitor do lado de mim.

Pesquisador: Você ia falar?

Participante: Então, eu ia falar que eu moro aqui há pouco tempo né, então eu ia falar assim que era uma coisa que estava faltando, que eu não sabia que tinha, dai ela acabou de comentar que tem, mas é uma coisas assim que faz falta, porque as vezes a gente fica em casa e não faz nada e falta isso ai, porque nós já moramos afastadas da cidade.

Participante: Na ... tem até hidroginástica.

Participante: Então né, eu mesmo já senti falta disso, por mim, pela minha mãe, meu marido que gosta de fazer, né.

Pesquisador: É importante ter as atividades e que elas sejam no bairro.

Participante: Ela é longe, onde elas estão falando, para quem é daqui do Aracy I é longe, é lá na entradinha do Antenor.

Participante: Ixe, tem muitas mulheres daqui que vai.

Participante: Então tem, mas tem muitas pessoas que não gostam de andar, eu mesma não aguento ir até lá.

Participante: É bem distante daqui na verdade.

Participante: Tem muitas daqui que vai.

Pesquisador: E essa, essa coisa que a gente estava falando de ter uma ligação, de ter uma mensagem para vocês, vocês acham que seria legal que essas mensagens sejam para estimular? Ou vocês também gostam de uma mensagem que elogia? Por exemplo “você está fazendo bem”, “Você está cumprindo os exercícios”, também liga para elogiar.

Participante: É lógico.

Pesquisador: Vocês acham que isso anima?

Participante: Eu acho bastante.

Participante: Eu acho.

Participante: Elogio cabe em qualquer lugar, em qualquer hora.

Participante: Ajuda bastante.

Participante: É, ponto também, sabe? Tipo, o grupo é de 20 pessoas, aquelas que não faltam, sabe? “Oh fulana, Oh dona Maria, você está de parabéns porque você cumpriu a meta e não teve nenhuma falta”, aquela amiga que faltou: “Oh amiga, tudo bem se você faltou talvez por algum motivo de doença ou por algum motivo da força maior, mas”. Até um brinde, alguma coisa, uma brincadeira, sei lá, alguma coisa assim para incentivar as pessoas, isso é muito bom.

Participante: E ai que a gente consegue muita amizade com as pessoas, eu acho muito bom.

Participante: Então, se tivesse um local, certinho, a gente já sabia onde tinha, ai ia lá e animava mais de fazer, “ai tem” nós vamos, “quais são os dias?”, “quais são os horários?”, ai a gente já sabe onde tem e é só ir até lá. Mas não tem. Ninguém oferece nada para a gente.

Pesquisador: O senhor não vai palpitar?

Participante: Eu só estou ouvindo

Pesquisador: Risos. Não, pode falar, nós queremos saber sua opinião.

Participante: Eu acho que tudo qualquer profissão da vida você tem que ter um seguidor..., pelo menos nos começo tem que ter até para te orientar para você não começar a fazer o exercício errado, porque as vezes você vai fazer um exercício para melhorar e acaba travando outra coisa.

Pesquisador: Entendi. E essa coisa da mensagem, o senhor gostaria de receber uma mensagem?

Participante: Eu já sou uma pessoa que já pensa diferente, eu acho que é minha responsabilidade e eu tenho que ter a minha meta da minha responsabilidade, de eu saber aquele dia da minha responsabilidade..., estar todo dia. Eu me sinto até mal, até na minha casa eu falo com a minha mulher e ela fala “faz isso” para uma coisa que eu já ia, ah, risos, aquilo me irrita, eu falo para ela “não me manda fazer aquilo que eu já vou fazer”. Toda a minha vida foi assim.

Participante: Eu já penso o contrário, eu gosto. Eu acho que está me incentivando, então eu gosto.

Pesquisador: Entendi. E durante, por exemplo, se você está durante a atividade, vamos supor que sua atividade é fazer caminhada, ai durante a caminhada, vamos supor, você recebe uma mensagem “É isso ai, você está caminhando em um ritmo bom” ou então “Aumenta seu ritmo”. Vocês gostariam durante a atividade receber a mensagem?

Participante: Se não fosse atrapalhar. Porque talvez se você esteja no pique da caminhada você não vai parar para olhar o celular.

Pesquisador: É ai teria que ser de uma forma dinâmica.

Participante: É, ai devia de, é, essa professora ou instrutor, não sei como vai chamar a pessoa, como chama a pessoa que vai guiar a gente?

Pesquisador: Pode colocar o nome que quiser, seria o celular, a distância.

Participante: Ah, seria a distância.

Pesquisador: Mas você pode imaginar como se a pessoa estivesse ali.

Participante: Eu na verdade quando vou fazer caminhada eu não ando com celular, eu quero andar despreocupadamente, Risos, então eu não ando.

Participante: Risos, então não ande comigo, porque senão nós vamos bater papo e não vai dar certo. Risos. Você perde aquele pique, se você faz calada você pega um pique e se for batendo papo, entendeu, ai sai fora do ritmo.

Pesquisador: Por que? Porque você tem medo que ele seja roubado ou porque você não quer receber ligação, não quer ter que atender?

Participante: Não, eu quero fazer minha caminhada, quero fazer minhas atividades.

Participante: Ela quer deixar tudo lá.

Participante: Sossegada, eu estou ali para fazer minhas atividades.

Pesquisador: É um momento seu consigo mesma?

Participante: É, então eu deixo o celular em casa.

Pesquisador: Entendi.

Participante: Eu mesma levo sempre o celular, mesmo dentro de uma igreja.

Participante: Eu mesma não levo.

Participante: Eu levo porque as vez acontece algum problema com a filha, que não sou só eu, sou eu e minha filha, que nem aconteceu essa semana, ela passou mal na escola, quando deu nove hora a hora que levantamo para ir embora do culto o celular tocou, olhei e número fixo, não tenho parente com número fixo e nove hora da noite não é posto de saúde. Olhei, atendi e já sai andando com o celular dentro da igreja para atender, era a diretora pedindo para socorrer a minha filha que tava vomitando sangue, tava passando mal, eu sai correndo para socorrer ela. Então eu acho que o celular é bom nessas hora, se quebra um carro, que nem já aconteceu

comigo várias vezes de quebrar um carro, eu sou sozinha, ligo para um, ligo para outro, ligo para outro, sabe é muito importante.

Pesquisador: E essa mensagem que vocês receberiam, vocês preferem que elas venham em forma de texto, escrita, mensagem escrita ou que seja alguém falando, uma voz?

Participante: Falando é melhor.

Participante: Que escreva, porque a escrita chama muita atenção. Risos.

Pesquisador: Você prefere qual?

Participante: Assim que escreva, porque fica mais né.

Pesquisador: E o senhor não prefere nenhuma? Que te deixem. Risos.

Participante: Risos.

Participante: Porque se for para incentivar, para ajudar é bom né.

Pesquisador: E a senhora, prefere que jeito?

Participante: No celular? Ah, para mim... meu celular é simples, só recebo ligação dos meus filhos, faço para eles, só isso. Não sei mexer com esse celular não.

Pesquisador: Entendi.

Participante: Mas é bom né.

Participante: Eu gosto dela falada, porque falando parece que a pessoa está falando realmente de você.

Participante: É.

Pesquisador: E, e falando mais dessa coisa do grupo que vocês estavam falando, né, que vai e o grupo te estimula, etc e tal. Quando vocês, assim, estão fazendo atividade no grupo vocês gostam de fazer, assim, um contra o outro, de competir, ver quem é melhor, quem vai ganhar ou vocês gostam de fazer de colaboração, igual uma gincana, se as pessoas fizerem juntas ai ganha melhor?

Participante: Colaborar.

Pesquisador: Colaborar?

Participante: Porque olha, para você ver, ela acabou de falar que ela já fez e já ficou para trás, porque tem mais pessoas de mais velho e mais novo

Participante: É, porque eu faço tudo no meu limite.

Participante: Ai vai ficar para trás, de você fazer e ele fica para trás, então vamos colaborar? Vamos fazer todo mundo junto? Todo mundo no mesmo ritmo, não é não? Vamos fazer todo mundo no mesmo ritmo, não é nao?

Participante: Ai não é justo eu ganhar e o outro não.

Pesquisador: Vocês gostam mais de colaborar então?

Participante: É.

Participante: Ah, com certeza, vai deixar o outro para trás, porque se nós estamos em um grupo é em um grupo.

Pesquisador: E o senhor?

Participante: Eu para falar a verdade estou pensando nisso pela primeira vez.

Pesquisador: Não, não tem problema, mas assim, se o senhor fosse fazer atividade física, o senhor gostaria de mais de fazer em grupo ou sozinho?

Participante: Ah, tem que ser em grupo, porque, é

Participante: Eu acho que tinha que ser dois grupos amiga, aquela que por exemplo, a moça jovem que nem você não vai acompanhar eu que estou com falta de ar e não...., ai eu vou estar atrapalhando a sua caminhada, porque você é jovem e está na ativa de, no pique de fazer rápido, entendeu? E eu já vou estar no pique de fazer menos, entendeu? Ah, eu, eu, falando de mim, quando eu fui fazer eu via as meninas no pique eu quis tentar.

Pesquisador: Então o que você está dizendo é que tinha que ser mais individual assim, nesse sentido

Participante: É, porque

Pesquisador: O progra, o treinamento tinha que ser específico para cada pessoa?

Participante: Mas é

Participante: É, porque por exemplo, eu e a senhora ali nós somos mais de idade

Participante: Não, eu estou entendendo.

Participante: Talvez eu não consiga andar igual ela, talvez ela não consiga andar igual eu.

Participante: O que ela quer explicar, quer dizer, o grupo, eu vou explicar melhor para entender, faixa etária assim para as pessoas mais velhas e para as pessoas mais velhas, o mais novo e o mais novo, porque as vezes meus filhos....

Participante: Nós temos isso ai.

Participante: Mas as vezes uma pessoa mais velha andar melhor que o mais novo também.

Pesquisador: Mas se fosse para fazer uma atividade, o senhor prefere competir, ver quem é melhor, igual por exemplo quando é futebol um vai jogar contra o outro e um dos dois vão ganhar, né, agora peteca, peteca não tem vencedor, caiu a peteca todo mundo perde, entendeu? Vocês preferem, vocês entenderem a diferença? Risos.

Participante: Eu não sei nem como é o nome, se é grupo ou como é que é, mas eu acho se é um programa pra, vamos supor terceira idade, que é o nome mais usado que a turma fala, terceira idade, tinha, tem que ser não competição um com outro, ser coletivo,

Pesquisador: Colaborar um com o outro? Um ajudar o outro?

Participante: É, tem que ser coletivo, senão não tem nem sentido.

Participante: Ele falou assim “dividir os grupos mais velhos e mais novos”, olha, os meu marido tem 66 anos e eu não....

Participante: Os velhinhos estão bem...

Pesquisador: É, tem que dividir os sacudidos e os não sacudidos.

Participante: Mas é isso o que eu ia falar, às vezes tem pessoas que tá mais velhos que a gente e tá melhore de saúde que a gente.

Pesquisador: Tem que ser uma coisa mais individual do que por faixa etária, né.

Participante: É, tem que ser mais individual, cada tem que fazer o que dá para fazer.

Pesquisador: É igual roupa né? Tem roupa que é de liquidação, é, tem roupa que é de fábrica e tem costureira que faz a roupa só para a pessoa, não tem?

Participante: Sobre medida.

Pesquisador: Então vocês prefeririam, por exemplo assim, que o treinamento de vocês fosse feito só para você ou fosse igual para todo mundo?

Participante: Ah, se for em grupo tem que ser no grupo, mas se for treinamento para cada um no celular cada um vai ter a opinião de fazer onde quiser.

Pesquisador: Se for a opinião.

Participante: Por exemplo: a senhora pode fazer 9 horas da manhã e eu posso fazer as 11 ou ela pode fazer as 7 e eu só posso fazer as 9.

Participante: Escolher horário.

Pesquisador: Isso é bom, poder escolher o próprio horário.

Participante: É, isso ai é bom.

Participante: É bom.

Participante: Eu tenho muita dificuldade em estar 7 horas da manhã em qualquer lugar.

Participante: Eu já tenho, eu já se eu pudesse 7 horas da noite já fiz tudo na minha vida. Eu fico bem.

Participante: Mas então, mas eu tenho problema de saúde, tem dia que eu não durmo a noite inteira, vou dormir quatro, cinco horas da manhã, ai eu quero dormir até nove hora.

Participante: O meu problema é levantar tarde.

Pesquisador: Então além do horário, do nível de treinamento, outras coisas também seriam importantes para escolher cada um né, para cada um. E, e vocês acham que ajuda nesse cada um ter uma, uma, um objetivo, uma, uma coisa que você tem que alcançar? Por exemplo assim:

vamos supor que você anda um quilometro, ai você tem o objetivo “não até daqui uns 2 meses eu vou conseguir andar mais um quilometro, dois”.

Participante: Ah, sim.

Pesquisador: Isso ajuda vocês? Estimula?

Participante: Estimula.

Participante: É assim, você está em uma caminhadinha de, dali vamos supor, um quilometro e você faz tempo que não faz, suas pernas já vai se

Participante: Eu durmo com dor e acordo com dor todo dia, tomo todo dia remédio para dor.

Pesquisador: E essa coisas que a gente estava discutindo de se competir ou de você colaborar, vocês gostariam, por exemplo assim se fosse no celular teria como outras pessoas verem, por exemplo ou o neto de vocês ou outra pessoa do grupo ou filhos?

Participante: Legal

Pesquisador: Vocês gostariam de compartilhar com outra pessoa, de mandar para outra pessoa?

Participante: Eu gostaria.

Participante: Eu também gostaria.

Participante: Sim.

Participante: Eu gostaria.

Pesquisador: Você tem Facebook?

Participante: Tenho.

Pesquisador: Você gostaria por exemplo de conseguir postar em um grupo fechado no face quais são

Participante: Eu gostaria.

Pesquisador: Ai por exemplo tem você e tem ela, você fala “ah consegui cumprir meu dois quilômetros de hoje”, ai ela posta “ai, eu também”, ai vocês gostariam disso assim?

Participante: Isso é bom, porque se tiver outras pessoas de outras cidades de outros lugares, mesmo que não seja do grupo ou que participe de algum outro grupo, fazer caminhada.

Participante: É, tem o jovem, criança, as crianças..., os jovens entendem de computador, televisão

Pesquisador: Entendo, você gostaria que esse grupo fosse de diferentes idades para conseguir estimular as crianças?

Participante: É

Pesquisador: Interessante isso.

Participante: Porque tem muita criança também que falar a verdade para você viu, precisa.

Pesquisador: Precisa.

Participante: O computador, a televisão

Participante: O computador e a televisão está deixando muito a pessoa obesa, principalmente as crianças. Meus netinhos estão tudo gordos, eu comecei a dar risada deles, falei para eles "antes eu pegava aqui e chacoalhava" agora "a vovó vai pegar aqui em vocês e chacoalhar", ele estava com 3 dobras aqui, gordinho, tem 12 anos só.

Participante: Ficam na rua soltando pipa, perigoso, rede elétrica, eles não se importam, pulam no muro do vizinho e tem um cachorro ali, não sabe se tem uma aranha ou alguma coisa. Então se tivesse alguma coisa para oferecesse a eles também, se ocupar, fazer alguma coisa assim, eles não estavam na rua também né, fazendo o que não devem, vendo o que não devem também, com certeza,

Participante: Mas eles se interessam menos do que nós.

Participante: É, porque os pais não incentivam.

Participante: Eles se interessam menos, porque no caso meu memo eu estou interessada porque minha saúde está muito debilitada, então, eu vou ter que achar um tempo para fazer isso.

Participante: E você vai gostar.

Pesquisador: E, e a se é, por exemplo assim a gente estava falando né se colocar uma meta ou um objetivo facilita né, falar eu vou fazer. Como que vocês fazem isso? Quais que vocês se colocam as vezes? Vocês têm alguma experiência de falar "não, eu vou fazer tal coisa" ou não? Vocês já tiveram essa experiência?

Participante: Ah, tem que fazer igual eu fiz agora "Eu não posso, mas eu vou largar tudo e vou i", porque se o cê fica "nossa mas minha filha está lá com dor de estômago e o namorado dela está em casa hoje", "nossa ele vai ficar bravo de mim tirar ela da cama para levar lá olhar os milho meu para trabalhar". Não, eu cheguei nela e falei "filha, eu preciso de você agora", "para onde", "ficar no carro vendendo milho, porque os milhos são perecíveis e eu preciso ir na reunião agora e eu não quero faltar dessa reunião, porque se a moça que foi, (aquela moça ali né), ela foi até minha casa, a minha casa lá embaixo, quase no meio do mato, se ela fez o sacrifício de ir até a minha casa é por algum interesse de alguma coisa de bom para a gente, entendeu? " então eu falei "a gente tem que vir atrás também", a gente tem que tirar um tempinho nosso.

Participante: A gente tem que ter um tempinho para a gente. Às vezes a hora que a gente está vendo assim uma novela, alguma coisa banal, a gente ocupa com alguma que vai melhorando nossa vida, nossa saúde.

Participante: Eu tiro.

Participante: Né.

Participante: Seis e meia da manhã, agora ele está meio que sem poder andar e tudo, mas seis e meia eu tô saindo para minhas atividades, eu penso “quem gosta de mim sou eu, eu me amo, então eu tenho que fazer aquilo que é bom para minha saúde”.

Participante: Está certinha.

Participante: O serviço a hora que eu voltar eu faço ele

Participante: E você vai ser a minha incentivadora.

Participante: Risos.

Pesquisador: Risos.

Participante: Risos.

Pesquisador: Olha ai, já tem a coisa da colaboração em grupo.

Participante: Quando ela falou “já vou, faço”. Eu falei “Opa”.

Pesquisador: E vocês acham, por exemplo assim

Participante: Pegou a pessoa errada, porque ai ela quer que a pessoa, eu tô deitado quieto e ela quer que eu ponha o pé na cabeça, todo dia, risos.

Participante: Olha que beleza se eu tivesse alguém para fazer isso no dia comigo.

Participante: Oh, sacrifício né, devia de ser tão pouco, risos.

Participante: Doida, Risos.

Pesquisador: Sua própria treinadora.

Participante: É.

Pesquisador: Vocês gostariam, assim por exemplo, que quando vocês colocassem esse objetivo “Ah essa semana eu consigo andar só um quilômetro, a partir do mês que vem eu quero conseguir andar dois quilômetros” e ai quando você conseguir andar dois quilômetros você ganhasse 100 reais.

Participante: Hum.

Pesquisador: Vocês gostariam? Vocês acham que isso estimula?

Participante: Ai sim, risos.

Participante: Risos. Dinheiro.

Pesquisador: Risos.

Participante: Risos. Dinheiro.

Participante: Ai até eu que sou besta já saia caminhando.

Participante: Dinheiro a gente ia andar até cinco quilômetros.  A turma ia fazer.

Participante: Não sei se você conhece o Antenor Garcia, a gente já consegue ir do Antenor Garcia três quarteirões depois da linha do trem, eu e mais duas.

Participante: Qual linha do trem? Fica para que lado?

Pesquisador: E, e o que que isso estimula assim? Você sente alguma recompensa? O que você sente de benefício?

Participante: Eu sinto assim bem para minha saúde, para minha cabeça.

Participante: Ela se sente uma vencedora. Risos.

Participante: Risos.

Pesquisador: E se ao invés do dinheiro fosse, por exemplo assim, um trofeuzinho, uma figura de um trofeuzinho escrito "você venceu", também era legal?

Participante: Ah, melhor. Isso, um troféu significa assim, é uma, uma forma de vencer.

Participante: É.

Participante: É, o troféu se guarda, o dinheiro se gasta. Dinheiro faz bom? Faz bem

Pesquisador: Pois é, vocês gostariam de ganhar um presen, melhor um presente que guarda vocês acham? Uma coisa que fica registrado, que guarda.

Participante: É, de você poder falar "isso aqui foi de que eu consegui ganhar uma vez"

Participante: Eu já não acho que nem é bem um presente, é a pessoa se sentir bem, vou dar um exemplo de mim para você, eu morava no cruzeiro e as vezes eu vinha do centro para o cruzeiro apé. Quando eu mudei para aqui para o Antenor, eu tenho dois filhos

Participante: Não, quatro.

Participante: É, mas falo dos homens, que fazem caminhada, e eles faziam caminhada

Pesquisador: Dois filhos e duas filhas?

Participante: É, e eles falavam "o pai não guenta", só que eles não sabia que eu caminhava, é, às vezes nóis saia do Antenor e ia até o Botafogo eles achavam que eu não guentava subir a subida lá, mas eu falava "eu guento" porque eu estava acostumado a fazer.

Pesquisador: Entendi, então o senhor está dizendo assim, por exemplo, que se, é, mostrar que você é capaz para seus filhos?

Participante: Não, mas eu já fazia já.

Pesquisador: Risos, isso foi bom.

Participante: Eu tenho um filho que faz caminhada de domingo só que ele vai de carro, ele vai fazer lá enfrente o SESC, do passeio para lá.

Pesquisador: Meu pai ri de mim que eu faço isso também.

Participante: Ele vai de carro para fazer a caminhada dele, risos.

Pesquisador: Eu vou até onde eu vou caminhar porque eu acho difícil chegar lá.

Participante: É.

Pesquisador: Tendeu? Às vezes a rua é perigosa, não tem árvore, ai eu acho difícil, então eu vou de carro até onde eu quero caminhar. Só que é esquisito se você for pensar, não faz sentido.

Participante: Risos.

Participante: Se você for caminhar sozinha, se você for caminhar em qualquer rua, não é legal.

Pesquisador: Não é legal, mulher sozinha caminhando.

Participante: Se você for caminhar numa quadra que já é da turma fazer caminhada você sempre encontra alguém, se vai para cá, a outra vai para cá, ai se já, parece que já

Pesquisador: É, verdade.

Participante: Domingo ele pega o carro e vai.

Participante: De qualquer jeito aquilo se torna uma competência mesmo que a gente não queira, sabia?

Pesquisador: É.

Participante: Se trata se tornando.

Pesquisador: Uma competição né.

Participante: Arriscar muito também é ruim.

Participante: Não, nada que prejudique a saúde.

Participante: Não, não, não.

Pesquisador: Tá certo, agora a Pesquisador vai mostrar para vocês um ap, um programa de celular que foi feito para ajudar as pessoas a contar os passos que elas dão e ai ele conta os passos, ela vai mostrar umas funções que ele tem, ele conta os passos. Ai eu queria que vocês olhassem a cor, como que aparece os números, o que ele faz e o que que ele não faz, porque depois a gente vai fazer uma atividade em que vocês vão dar palpites, se vocês tivessem que construir o produto de vocês como que ele seria, o que ele teria de diferente desse, o que ele teria igual e o que que, o que que esse tem mas não ia ficar tanto assim, que vocês querem que muda, entenderam?
