# Supplementary material for: Tailoring digital apps to support active ageing in a low income community
Source: PLoS One. 2020 Dec 10;15(12):e0242192. doi: 10.1371/journal.pone.0242192 (PMC7728168; doi:10.1371/journal.pone.0242192)
Supplement: S2 Transcript — (PDF) [file pone.0242192.s006.pdf]

GRUPO SUFICIENTEMENTE ATIVOS

ENGAJADOS DIGITALMENTE

Apresentação Pesquisador workshop PACER

Pesquisador: Então gente, é basicamente isso que compõe o aplicativo. Vocês acharam interessante?

Participante: Achei legal.

Participante: É

Pesquisador: Gente, esse que ela mostrou para vocês é só para vocês terem uma ideia, como vocês não usam muito o celular pra vocês saberem o que existe, né. Mas assim, agora na segunda parte vocês vão olhar ele de perto, poder mexer nele, e poder falar o que vocês quiserem, daí vocês vão falar para nós o que vocês gostariam que mantivesse, continuasse no aplicativo, que foi bom, o que vocês gostariam que tirasse, ou o que vocês gostariam que tivesse de diferente que não tem, ta? A opinião de vocês é o que importa, não importa se tiver que desfazer o negócio e fazer tudo de novo.

Pesquisador: É pra vocês fazerem algo com a cara de vocês, com a cara do bairro de vocês, do Aracy, vão poder tirar fotos do espaço

Participante: Como se fosse uma televisão mostrando ne?

Pesquisador: Isso. É para vocês transformarem isso para que tenha a cara do bairro de vocês

Pesquisador: Então eu vou pedir para vocês se levantarem, sentarem nesses dois grupos que tem, separa mais um pouco meninas, põe lá na ponta, a Pesquisador sabe quem vai em cada grupo, então Pesquisador cê fala pra eles os grupos

-organização dos grupos separadamente-

Pesquisador: Esse aqui é o aplicativo que comentamos com vocês. Todo mundo consegue ver? Se eu colocar assim fica melhor?

Pesquisador: Cada um pode segurar um pouco

Pesquisador: Aqui marca os passos, né, no caso aqui demos 1133 passos aqui só hoje. Dona Participante?

Participante: É

Pesquisador: Clica ali. Isso, aqui, ó.

Participante: Aqui né?

Pesquisador: Uhum. Então, aqui quando clica nos objetivos a gente pode selecionar o que a gente quer fazer. Escolhe um para a senhora dar uma olhadinha. Pode colocar peso, altura, montar seu perfil

Pesquisador: O senhor quer por um objetivo?

Participante: Quero por pra emagrecer

Pesquisador: Pode por

Participante: Aonde que é?

Pesquisador: O senhor clica ali ó. Qual a modalidade dessas da lista o senhor quer

Participante: Eu quero caminhada

Pesquisador: E a quantidade de passos o senhor gostaria?

Participante: Vinte mil passos

Participante: Exagerado! *risos*

-Início da atividade na cartolina e post-it-

Pesquisador: Agora nós vamos começar a atividade que é o seguinte, o que nós temos que pensar? O que eu gostaria que mudasse, o que eu gostaria que continuasse do jeito que está, ou seja, manter, e o que eu gostaria retirar, e o que gostaria de por que é novo e não tem. Aí por exemplo, vou por aqui o que eu gosto. Gosto que marca tudo e monitora, então vou escrever aqui “marcar tudo”, tá? E colo aqui nessa parte de continuar, pronto! Então cada um vai ganhar um desses, nós vamos ajudar vocês a escrever, não se preocupem, pode desenhar também, nós adoramos desenhos! Isso Pesquisador, ajuda elas aí.

Pesquisador: A senhora por exemplo, tem algo que você gostaria de tirar, continuar ou mudar? Você já tinha comentado que gostaria de comando de voz, lembra?

Participante: É, isso é verdade

Pesquisador: Então comando de voz e um aplicativo falado. Isso daqui a gente vai por lá no mudar, cola ali esse é o seu. Pra representar isso daí desenha uma boquinha falando os comandos, o que você acha?

Participante: Tá bom, esse é o meu

Pesquisador: Que mais gente... Vocês acharam interessante aquela coisa de um celular poder comunicar com o outro o que tá sendo feito em tempo integral, compartilhar com as outras pessoas, mandar para as outras pessoas?

Participante: Ah é legal, mas acontece que tem gente que não tem o celular para mandar, né

Pesquisador: Sim, mas quem tem seria legal né? Poderia fazer um grupo né

Participante: Sim, seria legal

Participante: É

Participante: Tem como comunicar com o lugar que estamos caminhando?

Pesquisador: Com a pracinha por exemplo?

Participante: É

Pesquisador: OK, vamos escrever isso. “Comunicar com a pracinha”. Se você quiser desenhar uma pracinha pra ilustrar, uma árvore... Pode colocar no seu também.

Participante: Concordo com o dela

Pesquisador: Também?

Participante: Sim. Comunicar com os lugares é muito melhor

Participante: Sim, tudo isso podia ser de graça né? Porque tem quem num tem como pagar, né

Pesquisador: Vou escrever aqui, “de graça”. Nós vamos por lá no mudar, porque esse é pago.

Participante: Êee assim não dá, quando é pago a gente não tem condição

Participante: É, mas compartilhar é bom também, incentiva o outro

Pesquisador: Vou escrever compartilhar

Participante: Sim compartilhar com um grupo

Pesquisador: Compartilhar com grupos, vou por aqui no manter porque o aplicativo já faz isso

Pesquisador: Qual o senhor colocou? Da praça?

Participante: Sim, coloquei da praça, também pra ser de graça

Pesquisador: Certo

Pesquisador: Agora me digam o que vocês gostariam que tirasse

Participante: Olha, eu concordo com tudo que tá lá, eu gostei

Pesquisador: Você não gostaria que mudasse nada então?

Participante É

Vocês gostam de fotos? Por exemplo, tirar uma foto e mandar pra ele de um exercício para copiar, o que vocês acham

Participante: É, eu gosto

Participante: Incentiva todo mundo né

Pesquisador: Ok, vou por aqui fotos e imagens, e vou colocar aqui

Pesquisador: Uma coisa pra tirar então, de acordo com o que vocês falaram, é ele ser pago, né?

Participante: É

Participante: É isso aí *risos*

Pesquisador: Que mais gente, e letras? Tá muito pequena? E imagem, as coisas tão muito pequenininhas?

Participante: Tá bão!

Participante: Sim ta bom

Pesquisador: Cês estavam enxergando bem?

Participante: Tava

Participante: Tá bom porque a letra é grande

Pesquisador: E as cores?

Participante: Eu gosto da cor que está

Participante: Tá bom sim

Pesquisador: Gostaram das cores? Então “manter cores” e “manter fontes”

Participante: Eu gostei do azul

Pesquisador: E aquela ideia que vocês tiveram no grupo de ter alguém falando? Em vez de ser assim um monte de número e frases.

Participante: É, também!

Participante: Sim verdade

Participante: É bão também, né

Participante: Podia por o doutor né

Pesquisador: Avatar do doutor Celebridade Brasileira?

Participante: Sim seria ótimo

Pesquisador: Vocês acham melhor que esse avatar fosse um médico ou da area da saúde, um treinador?

Participante: Melhor ainda né

Pesquisador: Incentivaria mais?

Participante: Incentivaria

Pesquisador: Quem falou que gostaria que o treinador acompanhasse sempre, ao longo de toda atividade? Acho que foi do outro grupo

Participante: Eu falei também

Pesquisador: Também né! Então vamo por aqui pro senhor “um acompanhamento mais contínuo, durante a atividade”, né?

Participante: É

Pesquisador: Gente tá ótimo. Agora vocês podem desenhar. Se vocês fossem criar um com a cara de vocês, como que vocês fariam? A Pesquisador vai ajudar vocês a desenhar. Cada um pega uma caneta.

-Pesquisador desenha os itens listados: compartilhar com outras pessoas, comunicar um celular com outro....-

Pesquisador: Gente inclusive vocês podem por nome no aplicativo de vocês

Participante: Pode ser igual o doutor Varella né

Pesquisador: O nome do aplicativo? Pode ser “doutor alguma coisa” né, o que será

Participante: Doutor Sabido

Pesquisador: Doutor sabido! Esse vai ser o nome então

-Pesquisador desenha o avatar do Celebridade Brasileira dentro do aplicativo, os sujeitos opinam como está ficando os desenhos-

-Pesquisador opina nos desenhos também, observando alguns aspectos simbólicos para os desenhos-

Pesquisador: Bom agora para finalizar vou escrever o nome de vocês aqui, componentes do grupo. Como é o seu?

Participante: Participante

Pesquisador: Com ‘p’?

Participante: Isso

Pesquisador: E você?

Participante: Participante, com ‘p’

Pesquisador: Hum ok, a senhora?

Participante: Participante.

Pesquisador: O seu é dona Participante né?

Participante: Sim isso

Pesquisador: O seu é Participante?

Participante: É Participante

Pesquisador: Participante. E o nome da senhora?

Participante: Participante!

Pesquisador: Todo mundo tá no grupo? Então tá bom

Pesquisador: Doutor Sabido é o nosso aplicativo, da boa alimentação e hábitos saudáveis

Pesquisador: Gente agora tem que apresentar o aplicativo de vocês pro outro grupo, o outro grupo vai apresentar o deles também. Quem vai apresentar? Se vocês estiverem com vergonha a Pesquisador pode ajudar

-Organização dos dois grupos em grande roda para apresentar os aplicativos criados-

Os grupos apresentaram os aplicativos uns aos outros, após isto foi realizado o coffee e encerrada a atividade.

NÃO ENGAJADOS DIGITALMENTE

Apresentação Pesquisador workshop PACER

Pesquisador: É pessoal, são essas coisas que tem no aplicativo, se vocês tiverem alguma dúvida vamos explicar melhor com vocês vendo, vocês gostariam?

Participante: Bem, eu não tenho celular

Participante: Eu gostaria sim

Participante: É

Pesquisador: O que você gostaria de por no aplicativo aqui?

Participante: Perda de peso

Pesquisador: Pode por

Participante: Põe pra mim caminhada

Pesquisador: Clica ali ó. Isso, ali

-Iniciada a atividade da cartolina e post-its-

Participante: Eu gostaria de manter a caminhada, contando os passos

Participante: Poe no manter o objetivo de perda de peso

Participante: Eu gosto de fazer ginástica na praça com aqueles aparelhos, daí compartilhar incentiva outras pessoas a usar os aparelhos

Participante: Pena que depois de umas horas fica perigoso né, não da pra praticar mais

Participante Olha, o que eu não gostei desse aplicativo é que tem que ficar andando com o celular pra fazer a atividade, não gosto de levar

-Iniciada a atividade de desenhar o novo aplicativo-

Participante: Desenha uma pessoa

Pesquisador: Como um treinador? Avatar?

Participante: Isso

Pesquisador: Ele fala pra vocês o exercício? Por texto ou mensagem de voz?

Participante Mensagem de voz!

Participante: Sim, mensagem de voz

Participante: Posso tentar desenhar uma pessoa comunicando o celular com outra?

Pesquisador: Pode sim, desenha aqui

Participante: Vou desenhar aqui também pessoas comunicando

Participante Eu não consigo desenhar, faz pra mim compartilhando imagens no grupo

Pesquisador: Desenho sim

Pesquisador: E o nome gente? Vamos pensar no nome do aplicativo com a cara de vocês

Participante: Podia ser grupo unido né?

Pesquisador: Perfeito

-Agora os grupos apresentam os aplicativos criados uns para os outros-

Após isso foi o coffee e a despedida.

GRUPO INSUFICIENTEMENTE ATIVOS

Apresentação Pesquisador workshop PACER

Pesquisador: Então é basicamente isso o aplicativo. Agora nós vamos mostrar para vocês. Vocês gostariam de ver?

Participante: Com certeza

Pesquisador: Agora nós vamos separar os grupos, para vocês darem os palpites no produto. Quem usa mais celular, vai ficar nesse grupo aqui, e quem usa menos o celular vai ficar no grupo de lá.

Participante: Entendi. Estou no menos

Participante: Eu também

-Separação dos grupos por engajamento digital-

GRUPO ENGAJADOS DIGITALMENTE

Monitora Pesquisador mostra o aplicativo no tablet para os idosos, mostrando novamente os recursos disponíveis.

Pesquisador: O aplicativo possibilita compartilhar o progresso das atividades com um grupo.

Participante: Sabe eu acho muito bom compartilhar, eu sempre compartilho o almoço com a minha irmã falo "olha aqui irmã meu prato de salada" porque ela gosta de comer fritura, tudo essas coisas, aí a gente tenta incentivar ela a comer mais salada, essas coisas

Pesquisador: O aplicativo mostra quantidade de passos, calorias, o tempo da atividade, ele marca tudo da atividade que foi feito na semana

Participante: Bem, você pode anotar aqui o nome do aplicativo pra mim? Daí vou mostrar pro meu marido

Pesquisador: Anoto sim, é Pacer.

Participante: Eu acho muito interessante, eu nunca tinha ouvido falar em aplicativo que marca a caloria... É isso né? Caloria, a quantidade de passos, o peso, ele acompanha o progresso ainda como você disse, olha só que coisa diferente.

Participante: Dá pra conectar com o facebook como você disse pra gente? Depois você ensina a fazer pra compartilhar?

Pesquisador: Claro

Pesquisador: Agora vamos começar a atividade, vocês vão escrever aqui no papel o que vocês gostaram no aplicativo, o que vocês não gostaram, tudo aquilo que vocês mudariam também.

Eu vou começar aqui pra vocês entenderem mais ou menos como que faz ta bom? Eu queria tirar o pagamento, porque ele tem uma parte que é paga e eu acho que poderia ser todo gratuito.

Participante: Ahhh, importante, assim a gente temos acesso

Pesquisador: Pode pensar em alguma coisa, como por exemplo, aquilo que você gostou de compartilhar nas redes sociais você gruda ali no manter, porque o aplicativo já faz isso.

Participante: Pode por tudo junto ou põe cada um em uma folha?

Pesquisador: Põe cada um em uma folha, melhor né

Pesquisador: Você tinha dito que gostaria de mensagens de incentivo né? O Pacer faz isso? Eu acho que não, então tem que grudar aqui ó. Escreve "mensagens ou áudios de incentivo", né?

Pesquisador: O que vocês preferem? Que essas mensagens de incentivo sejam escritas ou por áudio, voz?

Participante: Por voz!

Pesquisador: Então pode escrever aí, "mensagens de incentivo por voz e figuras" porque esse seria o formato né, voz e figura.

Participante: Eu acho interessante o seguinte, imagina que eu acordasse num dia com preguiça e não quisesse fazer nada, daí aparece uma pessoa chamando no celular "oi e aí vamos praticar uma atividade faz bem pra saúde" fazendo uma graça com a gente, é diferente né

Pesquisador: Uma pessoa chamando você diz um amigo seu ou um avatar?

Participante: Um avatar, uma pessoa que a gente não conheça

Pesquisador: Algum treinador?

Participante: Sim pode ser, bem

Pesquisador: Então vou colocar aqui no papel "treinador avatar"

Participante: Gosto quando incentiva também

Pesquisador: Vou por aqui

Pesquisador: Vocês acham assim, quando bate uma meta, receber uma premiação? Premiação de ponto, de recompensa, figurinha feliz, alguma coisa do tipo?

Participante: Prêmio cabe em qualquer lugar, fia

Pesquisador: Então põe aí, prêmio.

Participante: É porque a gente se sente realizado, né, é como se tivesse lembrando da gente. Sempre cabe né? Sempre cabe, né? É bem-vindo.

Pesquisador: E a cor, vocês acharam boa?

Participante: A cor do que?

Pesquisador: As cores do aplicativo

Pesquisador: As cores, as letras, se o tamanho é bom, tudo essas coisas

Participante: Aqui nessa parte eu acho que a letra precisaria aumentar, porque eu por exemplo uso óculos e não consegui ver direito. Então dificulta, porque por exemplo, eu vou fazer a atividade de óculos pra enchergar as letras no celular? Aí fica difícil né

Pesquisador: Então você acha que devia aumentar a letra?

Pesquisador: Aumentar a fonte, coloquei aqui.

Pesquisador: qual o outro aspecto que você gostaria de anotar?

Participante: Compartilhar né, foi o que eu gostei

Pesquisador: Vocês gostariam de outras medidas, além dessas que o aplicativo já tem?

Participante: Eu acho que deveria medir pressão

Pesquisador: Então vou por no manter as medidas que ele já faz e no mudar colocar outras pra aumentar o monitoramento.

Pesquisador: Pesquisador, agora podemos começar o re-desenho.

Pesquisador: Ok. Agora nós vamos desenhar o aplicativo, como que ele seria, vocês podem desenhar ou podem falar pra mim que eu desenho.

-Os itens escritos no post-it foram ilustrados na cartolina do re-design-

Participante: Olha, vou falar uma coisa pra vocês aqui. Esse debate, encontro que a gente fez aqui foi muito bom, é quase uma terapia, porque incentiva a gente de coisas que a gente já tinha vontade, mas precisava ainda de uma sacodida, entende? A gente tem vontade de falar, a gente tem vontade de fazer mas as vezes não tem como entende? E não tem por onde começar

Pesquisador: E nome gente, como vai chamar o aplicativo?

Participante: Amigos anônimos? Não anônimos não

Participante: Amigos Unidos!

Pesquisador: Amigos unidos tá excelente

Pesquisador: Ela falou pra poder escolher o horário pra fazer atividade, bota aí também.

-após isto, os grupos apresentaram os aplicativos criados uns para os outros, com os participantes em roda. Após, foi feito o coffee de despedida-

GRUPO NÃO ENGAJADOS DIGITALMENTE

Apresentação Pesquisador workshop PACER

Pesquisador: Vocês gostaram?

Participante: No meu caso sim

Participante: Eu não tenho celular fia, não sei mexer

Pesquisador: Vocês gostam da ideia de marcar os passos?

Participante: Sim

Pesquisador: E por objetivos?

Participante: Objetivo é bom pra tudo na vida né

Participante: Gostei desse de perda de peso que ela falou

Participante: Fazer a caminhada é muito bom, como aquela lá falou, mas pra mim é dicícil, eu ando bem devagarzinho porque já sou de idade, tenho dor no joelho e dor nas pernas. Mas eu gosto da caminhada, é muito bom

Pesquisador: Aqui nessa parte do aplicativo da pra ver a quantidade de passos na semana, da pra ver que dia andou mais. Você acha interessante isso?

Participante: Eu acho, faz um acompanhamento do que estamos fazendo, né

Participante: Eu acho.

Participante: Eu não tiraria nem acrescentaria nada, porque eu não conheço como mexe direito, mas tenho muita vontade de aprender

Participante: Outra coisa que ela estava comentando, eu acho que devia ser tudo de graça, porque a gente não tem condição nem para o aparelho, então fica difícil ne

Pesquisador: E um avatarzinho, um treinador, uma pessoa falando pra você na tela do celular o que você tem que fazer na atividade, vocês acham interessante?

Participante: Eu acho

Participante: Uma das coisas que eu gostei que eu vou falar pra vocês é a meta. Eu acho importante na vida, nos exercícios

Pesquisador: Vou escrever aqui

Participante: Eu gosto que ensina passo a passo, a gente aprende

Pesquisador: Que mais que vocês gostam?

Participante: Eu tinha um CD que era de treinamento, a voz da mulher ensinava o que tinha que fazer

Pesquisador: Você acha que mensagem de voz então?

Participante: Eu acho, com voz fica bom

Participante: Sim, porque tem gente que tem dificuldade com a leitura né

Participante: Verdade, eu não enxergo, a minha amiga além de não enxergar não sabe ler. Tem que ser uma coisa que pense na nossa condição, né

-Desenhar o novo aplicativo-

Pesquisador: O que vocês acham de desenhar um avatar?

Participante: Eu acho interessante, faz ele indicando como fazer o exercício pra não doer a coluna, dessa forma pra gente entender

Pesquisador: Vou desenhar ele indicando os exercícios

Participante: Desenha pra mim um grupo de pessoas caminhando juntas

Participante: Sim o que importa é a interação

Pesquisador: E o nome gente?

Participante: Bem-estar eu acho que é um bom nome

Participante: Eu gosto de atividade para a saúde

Pesquisador: Duas preferem atividade pra saúde, pode ser esse o nome então?

Participante: Sim

Pesquisador: Vou por o nome aqui então

Pesquisador: Qual o nome de vocês que eu vou por aqui?

Participante: Participante

Participante: Participante

Participante: Participante

Pesquisador: Depois vamos começar com a outra parte, vamos mostrar o aplicativo que a gente criou pra eles, e eles vão mostrar o que eles criaram pra gente, aí podemos ver, né, o que cada um fez, bonitinho

-apresentação dos aplicativos uns aos outros, após isto foi feito o café de despedida-
