# Supplementary material for: Tailoring digital apps to support active ageing in a low income community
Source: PLoS One. 2020 Dec 10;15(12):e0242192. doi: 10.1371/journal.pone.0242192 (PMC7728168; doi:10.1371/journal.pone.0242192)
Supplement: Attachment — Submitted filename: Response to Reviewers.pdf [file pone.0242192.s008.pdf]

Editor-in-Chief

PLOS ONE

Sao Carlos, September 9th 2020.

Ref: PONE-D-20-15049 “TAILORING DIGITAL APPS TO SUPPORT ACTIVE AGEING IN A LOW INCOME COMMUNITY”

Dear Editor,

We would like to thank you for handling our manuscript. We appreciate the comments and suggestions of all reviewers and respond to all of them in this letter. Regular font in blue are used to highlight the reviewer’s comments, while our response is in italic black font.

As a result of the corrections and following the suggestions, the new version of the paper is much improved, and we believe it will represent an important contribution to the field.

Respectfully yours,

Paula Costa Castro

Description of the changes made to the manuscript in accordance with the suggestions:

Academic editor

Recommendation: minor revision

1) Please ensure that your manuscript meets PLOS ONE's style requirements, including those for file naming.

The manuscript meets PLOS ONE's style requirements, which was carried out by the Overeaf platform. The files will be named according to the recommendations of Plos One.

2) We suggest you thoroughly copyedit your manuscript for language usage, spelling, and grammar. If you do not know anyone who can help you do this, you may wish to consider employing a professional scientific editing service.

The editing of the manuscript in relation to the spelling and grammar of the English language was carried out by two co-authors of the article, one who lives in England and has been a professor at the University of Surrey since 2005 and one who is a professor at the Hogeschool van Amsterdam (HvA) and professor at the University of Amsterdam (UvA) at the Institute for Informatics.

The main changes made by them were:

Abstract:

- *We exclude "In this way" before "maintaining physical activity levels during the life course is a motivational challenge".*

- *We exclude "Therefore" from the beginning of the objective.*

- *"The following procedures were performed: (i) baseline assessments; (ii) a focus group with physically active older people (iii) a focus group with insufficiently active older people (iii) design activities with both groups to re-design Pacer." has been modified to "The following procedures were performed: (i) baseline assessments; (ii) a focus group with physically active older people and a focus group with insufficiently active older people (iii) design activities with both groups to re-design Pacer."*

- *In the results of the abstract, the word "figures" was changed to "visual information"*

- "information exchange among users" has been changed to "sharing information among users"

Introduction:

-Line 31-33: We change from "Indicators of physical activity among Brazilians show that adults active in leisure, that is, who practice more than 150 minutes of moderate physical activity or 75 minutes of vigorous physical activity in the week, went from 30.3% in 2009 to 39.0% in 2019." to "In 2019, the percentage of Brazilian adults who were sufficiently active in leisure, which is, who practice more than 150 minutes of moderate physical activity or 75 minutes of vigorous physical activity in the week, was only 39.0%".

- Line 37-39: We change from "Motivating people for exercise has been one of the biggest obstacles to active life and reversing these numbers, as the behavior change process is not linear and requires individualized monitoring [14] [15]." to "Motivating people for exercise has been one of the biggest obstacles to active life, since the behavior change process is not linear and requires individualized monitoring [13] [14]."

Discussion:

- Line 598: We change from "The present investigation sought to identify..." to "The present investigation aimed to identify".

- Line 619: We corrected it from "the sample were" to "the sample was".

3) We note that [Figure(s) 1] in your submission contain [map/satellite] images which may be copyrighted. All PLOS content is published under the Creative Commons Attribution License (CC BY 4.0), which means that the manuscript, images, and Supporting Information files will be freely available online, and any third party is permitted to access, download, copy, distribute, and use these materials in any way, even commercially, with proper attribution.

-We removed the copyrighted images: In the case of Fig 1. a map from the PAUL application itself was inserted.

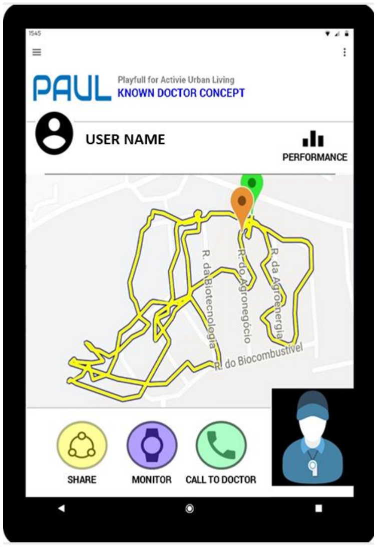

Fig 1. tif

4) We note that Figure(s) [2 and 4] in your submission contain copyrighted images. All PLOS content is published under the Creative Commons Attribution License (CC BY 4.0), which means that the manuscript, images, and Supporting Information files will be freely available online, and any third party is permitted to access, download, copy, distribute, and use these materials in any way, even commercially, with proper attribution.

-We removed the copyright images and inserted the functionalities instead.

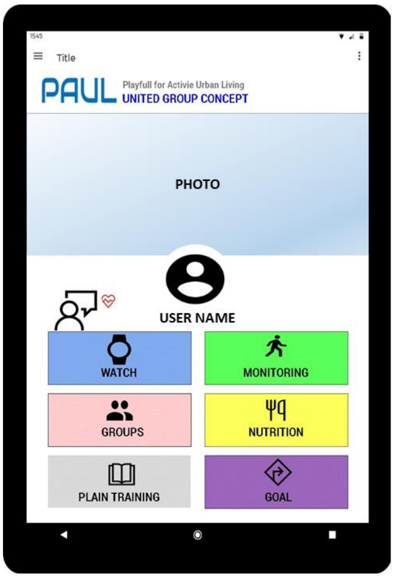

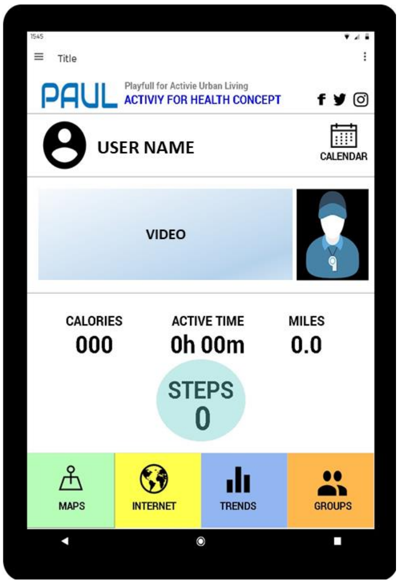

Fig 2. tif

Fig 4. tif

5) Thank you for sending us the data set underlying the results presented in your PLOS ONE submission. We notice that some of the information included in the data set may be potentially

identifying. Please ensure that the data shared are in accordance with participant consent and provide only the data that are used in this specific study. To ensure patient confidentiality, we would recommend removing the images containing names. Additional guidance on preparing raw clinical data for publication can be found in our Data Policy FAQs (https://journals.plos.org/plosone/s/data-availability#loc-clinical-data).

We remove the named images and insert "user name" instead. Below is Fig 3. as an example.

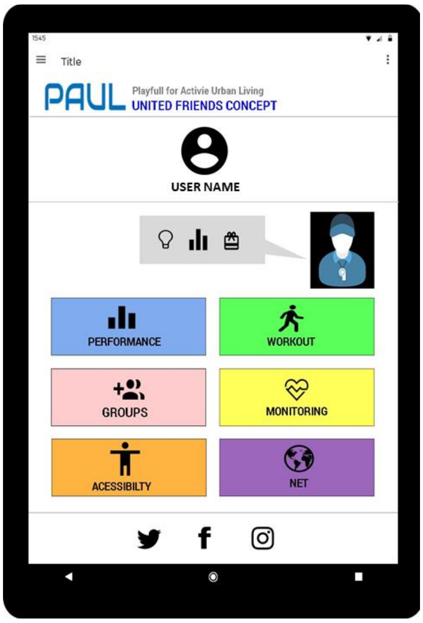

Fig 3. tif

6) Please include additional information regarding the survey or questionnaire used in the study and ensure that you have provided sufficient details that others could replicate the analyses. For instance, if you developed a questionnaire as part of this study and it is not under a copyright more restrictive than CC-BY, please include a copy, in both the original language and English, as Supporting Information.

Lines 126-137: We added "The questionnaire had five sessions: (i) sociodemographic information (such as name, gender, age, education, occupation and address), (ii) items from the International Physical Activity Questionnaire (lPAQ) [34] to evaluate the level of physical activity in Active Sufficiently and Insufficiently Active, (iii) items from the Behavioral Regulation in Exercise Questionnaire (BREQ 3) [35] about the individual perception about the practice of physical exercise, (iv) digital engagement information about (cell phone and smartphone use internet access, frequency of access, applications use and preferences, etc), (v) as well as semi-structured and open questions about the practice of physical activity (these

questions are related to the individual having practiced physical activity at some time in their lives, currently practicing or never having practiced). The complete questionnaire is available as additional material."

7) Please clarify whether the study was specifically approved by the IRB, and clarify how participants gave consent.

Line 83-87: About that we added "The research received ethics approval from the Federal University of Sao Carlos Ethics Committee on Human Experimentation (CAAE 81121317.3.0000.5504). Participation was voluntary and complete anonymity of participants was assured. All participants signed the Free and Informed Consent Form and received a copy of it in accordance with the guidelines and regulatory standards for research involving human beings."

Reviewer: 1

The authors are to be congratulated for taking on such an interesting an important research area to investigate, especially involving the vulnerable population of older people. While some parts of the manuscript were interesting, other areas need to be improved. We appreciate your contribution and hope the authors consider my feedback for enhancing their manuscript.

We appreciate the reviewer acknowledgment and try to answer all the questions below.

Introduction

1) Most of the background information lacks references resulting in lack of evidence for the authors claims. Revise and add the needed references throughout the introduction section to establish where those ideas came from.

All background information was reviewed, following an order for literary presentation. In this way, considerable changes were made throughout the structure of the introduction.

FIRST PARAGRAPH

Previously – Lines 2-8

Population aging is a worldwide reality, characterized by an increasing number of older people in the total population. This implies a change in the proportions of older age groups in the total population, primarily in developed countries. However, it is also happening at an accelerated rate in developing countries like Brazil. This transformation occurs due to the

demographic transition, which begins with declining mortality and birth rates, advances in medicine and better living conditions, causing significant changes in the age structure of the population [1].

<u>*Now - Lines 2-11*</u>

Population aging is accelerating worldwide. This phenomenon which occurred primarily in developed countries, is now happening in developing countries as well, with a gradual growth in the age groups of older people in the total of the population. This can be explained by the demographic transition, which begins with the decline in mortality and birth rates, accentuated by technological advances in medical sciences and by access to better living conditions [1] [2]. Despite these advances being considered an important achievement for the human legacy, especially for developing countries, such as Brazil (with 13% of aged population) [3], there is still a lack o studies that support social and public health policies that consider a contingent of the population that ages in a situation of social vulnerability [4].

<u>*SECOND PARAGRAPH*</u>

<u>*Previously – Lines 9 - 17*</u>

This demographic transition has increased the need for public policies for Healthy and Active Ageing with better quality of life. Considering that the dynamics of health in ageing are very complex and may lead the individual to a frailty process, morbidity compression has been considered one of the greatest public health challenges currently. The ageing process can be associated with the accumulation of molecular and cellular damage that lead to reduction of physiological reserves, increasing the risk for the development of disease and disability in old age. However, such changes do not rely only on chronological factors, but are strongly related to life-course behaviour patterns, environmental and socioeconomic factors and can be delayed or avoided [2]~~.~~

<u>*Now - Lines 12 - 20*</u>

Social vulnerability is defined in the literature as a multidimensional construct, in which behavioral, socio-cultural, economic and political patterns interact with biological processes throughout life [5]. These biological processes mean the accumulation of molecular and cellular damage that lead to the reduction of physiological reserves and increase the risk of chronic diseases (CD), such as cardiovascular and Type II Diabetes Mellitus [6]. These chronic conditions if not treated lead older adults to develop the frailty syndrome, in which

"compression of morbidity" is considered one of the greatest public health challenges [7] and is equally onerous to health economics worldwide[8].

<u>THIRD PARAGRAPH</u>

<u>Previously – Lines 18-26</u>

The practice of physical activity (PA) has been confirmed in the literature and defended in policies as a protection factor against these negative outcomes related to ageing. Scientific evidence that physical activity contributes to healthy ageing is well established. However, many older persons can be usually hypo-active, which increases the prevalence of syndromes related to inactivity, with increased risk of strokes, coronary diseases, diabetes, hypertension, among others conditions. At the same time, the psychological impact of low self-esteem, anxiety and depression worsens, with social consequences such as isolation and negative social image. This reduces social participation, which is one of the determining factors for Healthy Ageing [2].

<u>Now – Lines 21-29</u>

The chronic conditions compromise the performance of activities of daily living (ADL), such as feeding, dressing, bathing, sphincter control, sexual activity, self-care and mobility, with significant impacts on functional capacity and well-being in old age being very prevalent in older adults [9] [10]. However, these functions can be strengthened and rescued through regular and guided physical activity (PA), which has been shown to have a positive impact on the healthy aging process and to decrease the risk of chronic conditions [11]. More specifically, data from studies suggest that physically active individuals increase their chances of living a healthier life at older ages when compared to inactive ones [12].

<u>FOURTH PARAGRAPH</u>

<u>Previously – Lines 27 - 32</u>

Although the literature on ageing and PA shows the benefits of this practice to Healthy Ageing, the activity lifestyle profile of the Brazilian population is quite unique. Data from two national surveys – the National Diagnosis of sports and health Ministry research - indicated, respectively, that only 28.5% and 37.6% of Braziliansare active and 45.9% are completely

sedentary and that sedentarism is positively correlated with age (the older, the more sedentary lifestyle) [3] [4].

Now – Lines 30 - 36

Despite this, physical activity levels remain insufficient in older adults with low education in Brazil. In 2019, the percentage of Brazilian adults who were sufficiently active in leisure, that is, who practice more than 150 minutes of moderate physical activity or 75 minutes of vigorous physical activity in the week, was only 39.0%. Furthermore, the this practice tends to decrease with age: 49.4% in the range of 18 to 24 years and 24.4% in adults aged 65 and over. It also, which varies with schooling, from 25.8% (8 years of study) to 50.0% (with 12 years or more) [13].

FIFTH PARAGRAPH

Previously – Lines 33 - 38

In addition, the second research demonstrated that the higher the education level, the higher the level of PA, which can be a problem for the Brazilian vulnerable population with high rates of illiteracy. Given these numbers; interventions for motivating change behavior within a life course perspective, have to take account of the specifics of this population when making policy about practice of healthier lifestyle habits, essential for maintaining the quality of life [4].

Now – Lines 37 - 47

Motivating people for exercise has been one of the biggest obstacles to active life, since the behavior change process is not linear and requires individualized monitoring [14] [15]. Thus, identifying the behavioral state of physical activity can assist in the design of interventions. The Transteoric Model (TTM) for behavior change, for example, identifies five behavioral states of physical activity: (1) Pre-contemplation, who are neither active nor intend to change behavior. (2) Contemplation, individuals who are not physically active, but intend to start an activity. (3) Preparation, when they begin to develop activity slowly and gradually to desirable levels. (4) Action, those who already practice according to the recommended level for less than six months. (5) Maintenance, when the individual has been

practising for more than six month [16].

SIXTH PARAGRAFH

Previously - Lines 39 - 47

Unfortunately, it is difficult to motivate people to keep healthy lifestyle habits. The process of behavior change is not linear and relies heavily on the stage of change [5].
There are five behavioral states of physical activity: (1) pre-contemplation, individuals who are not active nor have the intention to change behavior; (2) contemplation, individuals who are not physically active, but intend to start an activity; (3) preparation, when they begin to develop an activity slowly and gradually to desirable levels; (4) action, those who already practice according to the recommended level for less than six months; (5) maintenance, when the individual already is practicing for more than six months [6].

Now – Lines 48 - 55

The application of the TTM for sedentary behaviors can provide crucial and in-depth information about participants' readiness to change their sedentary behaviors.
A recent approach to persuasion is the use of mobile health applications [17] [18].
Behavior change carried can be triggered and monitoring using smartphones that send motivational messages to an individual in order to shape their attitude or behavior [19]. Unfortunately, 50% of mobile fitness users stop using their fitness-tracking device after six months [20]. This is due to the lack of behavior change constructions that are necessary for long-term engagement [21] [22].

SEVENTH PARAGRAPH

Previously – Lines 48 – 53

In this way, different people usually need different motivation, since the existing barriers are quite different between the behavioral states. In a study with a socially vulnerable population, the barriers reported are related mainly to the inadequacies in environments, such as the lack of accessibility, guidelines, as well as the lack of company and the fear of falling. Keeping an exercise regimen for long periods under these conditions requires high levels of motivation [7].

Now – Lines 56 – 64

The uptake of mobile technologies in the market, as well as advances in their applications, offers a potential solution to support inactive individuals to improve their activity levels. More than half of the worldwide population now owns a mobile phone and, in most regions, at least 50% have access to the internet [23].In Brazil, there are already more active smartphones than people [24]. Despite the proven effectiveness of mHealth programs in supporting behavior change [25], there is still a dearth of research focusing on mHealth for people in vulnerable situations [26] [27]. Thus, it is important that strategies and interventions are adaptable to the needs of this population and designed through a process of co-creation.

EIGHTH PARAGRAPH

Previously – 54 - 59

In this scenario, the mobile applications have been shown to be a very innovative tool to encourage healthy lifestyle habits and the practice of PA, assisting in a range of functions to carry out exercises, such as the access to a coach who makes digital monitoring, encourages and assists via the mobile device. Thus, this kind of service can be very useful for public health programs, given the increasing rates of information and communication devices adopted by older people in Brazil [8].

Now – Lines 65 - 73

Co-design is a technique in which potential end users in partnership with other stakeholders and researchers work together in all stages of product development, from needs assessment, content definition, prototyping, testing and dissemination [28]. The dynamics offered by this technique allow for conceptual re-development and strategies based on the socio-cultural needs of different groups [29] [30]. Relatively new in the area of health, the concept has typically been used in projects to improve service to patients [28]. Nevertheless, it makes sense to consider this process for the development of interventions in mHealth research, especially now with the expansion of access to mobile technologies [31].

NINETH PARAGRAPH

Previously – Lines 60 - 69

With the massive proliferation of apps for smartphones, it becomes increasingly difficult to identify and evaluate their quality [12]. Currently, the use of mobile applications for health and wellness promotion has grown, but the mechanisms to evaluate the quality of the proposed service remain insufficient. The options are usually star ratings such as "very" or "not liked", as well as forums for discussion and feedback. Because they are subjective and sometimes anonymous by nature, they may come from suspicious sources, such as, the developer company [13].

Now – Lines 74 - 80

The market for this series of applications has spread rapidly; however, the evidence from research on the development and evaluation of these applications is still incipient in populations living in vulnerable situations. The main aim and contribution of the present study is to identify the requirements of older Brazilian people in a low-income community for mobile exercise apps. The co-design process enabled the development of product concepts with and for this population, to improve their physical activity in urban environments.

TENTH PARAGRAPH

Previously – Lines 77 - 84

Therefore, selecting applications based on popularity can produce little or no significant information about the quality of the product. On the other hand, applications built in partnership with the end-user bring knowledge about aspects related to a possible future rejection of the app, as well as elements that would make it more useful and usable, supporting long-term changes. The quality can be described as a function of: (1) content, (2) interface, (3) navigation, (4) structure and design and (5) singularity [14], assuming these as the essential components for a more robust feedback on the usability of the product [15].

ELEVENTH PARAGRAPH

Previously – Lines 85 – 92

Given the state of the art, we emphasize the importance of deeper studies on the preferences of end-users to co-design these apps. In this context, the present study aims to answer the following questions: which elements are indispensable for tailoring an application of physical activity for older socially vulnerable users? What are the strategies of gamification, preferences and problems that encourage or prevent the use of these applications? We propose to identify parameters and recommendations for the design of an application focused on end-users, based on the context and experience of people over 40 years of age in a high vulnerability group from Sao Carlos-SP, Brazil.

2) Paragraphs should include a minimum of 4 sentences. The authors should group short paragraphs that present the same topic into one paragraph.

1st par. Demographic transition/technological advances/development countries/study gap with socially vulnerable populations.

2nd par. Social vulnerability/biological process/chronic conditions/ public health chalanges.

3rd par. Activity of daily living/functionality rescue/ physical activity studies.

4th par. Physical activity levels in older and low education individuals in Brazil.

5th par. Motivation/obstacles/ behavior monitoring needs/ Transteorical Model.

6th par. Mobile health applications/motivation/user concerns/engagement/behavior chang).

7th par. Activity levels improvement/access to the internet/acess to the smartphones in Brazil/ user needs.

8th par. Co-design technique/co-design in mhealth researches.

9th par. Considerations regarding the goals of this study.

3) The background information about the effectiveness of co-designing apps is weak. Given that co-design an app is the main objective of this study, this evidence needs to be strengthen.

Background about co-designing apps

Co-design is a technique in which potential end users in partnership with other stakeholders and researchers work together in all stages of product development, from needs assessment, content definition, prototyping, testing and dissemination [27]. The dynamics offered by this technique allow for conceptual re-development and strategies based on the socio-cultural

needs of different groups [28] [29]. Relatively new in the area of health, the concept has typically been used in projects to improve _service_ *to patients [27]. Nevertheless, it makes sense to consider this process for the development of interventions in mHealth research, especially now with the expansion of access to mobile technologies[30].*

4) In the last paragraph, conclude with a comment about whether the study's aim was achieved.

A new sentence was introduced

The market for this series of applications has spread rapidly; however, the evidence from research on the development and evaluation of these applications is still incipient in populations living in vulnerable situations. The main aim and contribution of the present study is to identify the requirements of older Brazilian people in a low-income community for mobile exercise apps. The co-design process enabled the development of product concepts with and for this population, to improve their physical activity in urban environments.

Materials and Methods

1) What were the participants' exclusion criteria?

Lines 111 and 112: We added the phrase "The only criterion for the exclusion of study participants was living outside the indicated context (region)."

2) What was the guiding framework/theory used by the authors to collect, analyze, and interpret the study's data to co-design the app?

Lines 94-98: We added "This was to perform a creative co-design activity using the Focusgroup+ method [31]. This is described under Procedures section below, but essentially involves use of a conventional focus group to collect critical feedback on product concepts, before challenging sub-groups to re-design those concepts themselves."

3) When the authors state "A previous study database was randomized and 136 individuals who met the eligibility criteria were contacted…" Which study are the authors referring to? How were the individuals randomized?

Lines 99-107: We added "Databases from two previous studies were used in order to randomly select individuals over 40 years old to be included in the study. The studies were a controlled and quasi-experimental research for adults and elderly individuals in the locality of the present study [32]. And a cross-sectional study carried out with elderly people from the same region

[33]. Three undergraduate students contacted the individuals who met the eligibility criteria via phone in order to invite them to attend the two focus groups until a total of 24 participants were obtained (12 sufficiently active and 12 insufficiently active). To obtain this quantity in each group, 136 individuals were contacted. Their previous level of physical activity was verified in the database."

4) Specify who recruited the participants of this study (those who made the phone calls), who conducted the pre-interviews in the participants' home, who conducted the focus-groups and co-design activity? What are the qualifications of these researchers?

- *Lines 103-106: We add in the sentence the information of who recruited "Three undergraduate students contacted the individuals who met the eligibility criteria via phone in order to invite them to attend the two focus groups until a total of 24 participants were obtained (12 sufficiently active and 12 insufficiently active)."*
- *Lines 125 and 126: We added who conducted the pre-interviews in the participants' home "The pre-interviews made in the homes of the participants were carried out by undergraduate students, authors of this paper (Bonadio, Bet, Lorenzi, Paixao)."*
- *Lines 152-154: We added who conducted the focus-groups and co-design activity "They were facilitated by two PhD professors (Castro and Gomes) with help from Masters and Undergraduate students (Bonadio, Bet, Lorenzi, Paixao)."*

5) Can the authors include an introductory sentence with the stages of the focus group? Were these stages conducted in one focus group session or several sessions? It is not clear if the subsequent co-design activity in which the participants were sub-grouped into digitally engaged or not happened within the focus group or was separate activity.

Lines 145-151: We added "Two Focusgroup+ workshops were carried out in the following three phases: (1) general discussion of motivation to exercise following a short video, (2) introduction of a mobile exercise app called PACER ® for critical feedback, and (3) re-design of PACER by two subgroups. PACER was selected as one of the most popular exercise apps on the Google Play Store having pedometer and coach functions. All of these steps were performed on the same day, in a single workshop session lasting about 3 hours, with a short break between each phase."

6) Where were the focus group conducted?

Lines 151 and 152: We added "Sessions were held in an event hall of a well-known church in the region where the participants lived."

7) The sentence of ethical approval should be moved to the first sentence

Lines 83-85: We moved the sentence of ethical approval to the first sentence.

8) Data Analysis:

a. Add a section of data analysis at the end of the Materials and Methods section.

We added the data analysis section at the end of the materials and methods, as requested (see page 7, line 229 to 287).

b. What was the methods used for processing data prior to and during analysis, including transcription, data entry, data management and data coding?

The methods used for processing data prior to and during analysis were added in the data analysis section of the revised manuscript (see page 7, line 239 to 244):

"A set of communication analysis techniques was performed aiming to obtain, by systematic and objective procedures for describing the content of messages, indicators that allow inference of knowledge regarding production/reception conditions (inferred variables) of these messages points. This analysis was divided into (1) pre-analysis; (2) exploration of the material; (3) data processing and interpretation.".

c. State the authors or guidelines used by the authors to conduct the content analysis, not only the reference and the process by which inferences and themes were identified and developed, including the researchers involved in data analysis

d. What were the techniques used to enhance trustworthiness and credibility of data analysis?

We added the author/guidelines used on the paper on the "Data analysis" section on page 7, and the author involved in data analysis were listed during the revised version of the paper. The author that based the study methodology and analysis was Bardin (see page 7, line 239):

Reference: Bardin, L. (2011). Content Analysis. Sao Paulo: Edicoes, 70.

e. Consider changing "Simple statistical operations" for "Descriptive statistics". Include all the descriptive statistics used to analyze the quantitative data concerning the participants' characteristics (in addition to percentages and frequencies).

We changed this part in the revised version of the paper, and included all the statistics used to analyze the quantitative data:

"Descriptive analyses, such as median, minimum and maximum values, were performed to characterise the sample in age, gender, education, physical activity level, and digital engagement. The comparisons between the groups in the analysis were organised in order to: Compare the ACTIVES group in general, looking at their subgroups of engaged and not digitally engaged, also the INSUFFICIENTLY ACTIVE in the same modality. When then compared the subgroups by equal pairs, comparing active and insufficiently active with engaged-engaged and non-engaged-non-engaged. Comparisons for active-insufficiently active groups such as large groups were also designed." (see section "Data analysis" on page 7, lines 230 to 238).

9) Procedures:

a. Please, describe the "PAUL" project.

The PAUL project was described in the Procedures section (see page 5, lines 157 to 164) as follow:

"The PAUL project is a partnership between the Federal University of Sao Carlos (UFSCar), Federal University of Sao Paulo (UNIFESP), and the University of Amsterdam. This project has the main purpose to verify the effectiveness of an application for physical activity that has elements of gamefication and messages, and to change behavior by increasing the practice of physical activity. Active and healthy aging, motivation for physical activities in urban areas and applications for monitoring and training were also addressed. All participants were non-users of physical activity applications."

b. Add references to the following methodological action: "This reduced the risks of induction through the researcher's own posture and in the expression of the statements, as well as respect for the instructions for applying each instrument."

We understand that this phrase may cause confusion when the reader is reading. Thus, we deleted this phrase in the revised manuscript.

c. How “Active and healthy aging, motivation for physical activities in urban areas and applications for monitoring and training” were addressed during the focus groups? They received information about these topics from the researchers or these topics were addressed through the focus group questions?

The information about active and healthy aging were discussed during the focus groups. You can see this information on the focus group script, added as a supplementary document on the PLOS ONE system.

d. When the authors state “The participants gave their opinion, answered and discussed the issues before the group,” what does “before” mean? It is before the focus groups were conducted?

This phrase during the text was a mistake and was deleted in the revised manuscript.

e. The authors need to add references, explain and justify the “Keep, Loose and Change method” for Re –design. Why was that method chosen among the others?

This method is explained as following in the manuscript:

Page 6, lines 184 to 195: “In the second and third phases of the workshop, we introduced a commercially available exercise app called PACER Pedometer and Coach to the group for feedback and re-design. This technique was developed as an accessible way of doing co-design with a group of older adults who may be unfamiliar with new technology. Participants are shown a product concept for verbal feedback in the usual way. They are then split into subgroups and asked to consider what they would like to keep, lose and change about an existing product or product concept, as a precursor to deciding features of a new concept that they prefer.”

Page 6 and 7, lines 220 to 228: “After a break, the phase 3 re-design activity was then started, in which the participants considered what to Keep, Lose or Change about the app. This led to further design ideas for a new version of the app which was preferred by the whole group. Design ideas were collected through drawings and post it notes, where the participants drew and wrote down their ideas. They also made comments on themes, and ideas for tools which were video recorded.”

The reference used is: FROHLICH, David M.; LIM, Christopher Sze Chong; AHMED, Amr. Keep, lose, change: Prompts for the re-design of product concepts in a focus group setting. ***CoDesign****, v. 10, n. 2, p. 80-95, 2014.*

f. Authors should group short paragraphs that address the same topic.

This change was made throughout the revised version of the manuscript.

Results

1) An introductory paragraph should be included presenting the themes and sub-themes that emerged form the focus groups data.

The introductory paragraph was included in the beginning of the results section (see page 8, line 289 to 300), as following:

"The final sample of the study was composed by 17 participants, being divided into two groups: physically active (12 participants) and insufficiently active (five participants). Both groups were subdivided and classified according to the participants' digital engagement. The first group was composed of six people equally in the subdivision, while in the second, two were considered digitally engaged and three were not engaged. The main results found in this study point out that the development of physical activity applications for older users consider the following resources: free application, simple interface, motivational messages using audio and pictures, information exchange between users, multimedia input and sharing and user customisation. These insights can be important for designing intervention applications to improve healthy behaviour, considering a universal product, but still adapted, increasing the likelihood of adoption."

2) The results section needs to be better organized. If content analysis was used to develop the categories, why a Table 2 was inserted halfway of the result's section with only some of the categories?

We tried to organized better the result section during the revised manuscript. As a latex template was used to prepare the manuscript, the tables, specially Table 2, were distributed throughout the text but not necessarily after being cited, in order to optimize the space of the paper. However, table 2 has been changed from place to better understand the text (see page 8) in the revised manuscript.

3) The authors already stated in the methods section that a content analysis was used. No need to restate this in the results section.

This phrase was deleted in the revised manuscript version.

4) It is not clear why the authors inserted the Table 2 at the middle of the results section. If the authors used thematic content analyses for the presenting the results.

We already answered this question in this letter in "Results, topic 2".

5) Specify how many participants were in each sub-group.

This information was added in the manuscript as following: "The final sample of the study was composed by 17 participants, being divided into two groups: physically active (12 participants) and insufficiently active (five participants). Both groups were subdivided and classified according to the participants' digital engagement. The first group was composed of six people equally in the subdivision, while in the second, two were considered digitally engaged and three were not engaged." (see page 8, line 209-294).

Conclusions

1) I recommend to change the "Conclusion" subheading for "Discussion."

This change was made in the revised version of the manuscript on page 14.

2) Start this section by re-stating the purpose of your study.

We added the purpose of the study in the beginning of the discussion section (see page 14, line 598-602): "The present investigation aimed to identify the components necessary for the customization of an application to encourage physical activity, based on the wishes and experiences of users over 40 years of age living in a community of high social vulnerability in Brazil."

3) Add references to the following statement: "Focus groups have traditionally been used in market and design research.

The following reference was added to the revised manuscript: MURGADO-ARMENTEROS, Eva María; TORRES-RUIZ, Francisco José; VEGA-ZAMORA, Manuela. Differences between Online and Face to Face Focus Groups, Viewed through Two Approaches. ***Journal of Theoretical and Applied Electronic Commerce Research****, v. 7. n. 2. p. 73-86. 2012.*

4) Highlight what is new about your study.

This information was added in the revised manuscript, in page 15 and 16 (line 640 to 657), as following: "The dynamic approach adopted in this study allowed the participation of people with different profiles, which made the results more plural and more likely to be assertive. In addition, the facilitator's role was important in providing support and ensuring the involvement of all those present. Still, the methodology developed enabled the identification of several factors that must be considered in the process of creating an application to encourage physical activity. In this way, this study brings with it key elements for creating applications focused on the needs and desires of users, and innovates by bringing new findings to the scientific community, by conducting a study with a robust methodology, in a region of high vulnerability with participants over 40 years old and with a low level of education. The limitations of the study are that it enrolled only people over 40 years old in regions of high social vulnerability, so there was no inclusion of different individuals to compare with this population and check if there are different needs and specifications across other age categories or incomes. In addition, engagement and participation in the focus group was higher among active compare to the non-active group, it probably happened because the first group was more open to discussing physical activity itself. Other studies should find ways to encourage non-active people to engage in the debate and participate in the design of the app"

5) Study limitations are missing and must be established.

Lines 649-656: About the limitations of the study we added "The limitations of the study are that it enrolled only people over 40 years old in regions of high social vulnerability, so there was no inclusion of different individuals to compare with this population and check if there are different needs and specifications across other age categories or incomes. In addition, engagement and participation in the focus group was higher among active compare to the non-active group, it probably happened because the first group was more open to discussing physical activity itself. Other studies should find ways to encourage non-active people to engage in the debate and participate in the design of the app."

General recommendations

1) The manuscript requires to be proofread and edited for the English language.

The revision of the manuscript in relation to the spelling and grammar of the English language was carried out by two co-authors of the article, one who lives in England and has been a professor at the University of Surrey since 2005 and one who is a professor at the Hogeschool

van Amsterdam (HvA) and professor at the University of Amsterdam (UvA) at the Institute for Informatics.

2) This is a good study with unique population that does not get enough attention in the literature. I wish you the best and thank you for the opportunity to review your work.

Thank you! We hope that the modifications made to the manuscript are satisfactory and have addressed all necessary aspects.
