# Supplementary material for: Tailoring digital apps to support active ageing in a low income community
Source: PLoS One. 2020 Dec 10;15(12):e0242192. doi: 10.1371/journal.pone.0242192 (PMC7728168; doi:10.1371/journal.pone.0242192)
Supplement: Attachment — Submitted filename: Comments to the Authors Plos One.docx [file pone.0242192.s007.docx]

The authors are to be congratulated for taking on such an interesting an important research area to investigate, especially involving the vulnerable population of older people. While some parts of the manuscript were interesting, other areas need to be improved. We appreciate your contribution and hope the authors consider my feedback for enhancing their manuscript.

**Introduction:**

1. Most of the background information lacks references resulting in lack of evidence for the authors claims. Revise and add the needed references throughout the introduction section to establish where those ideas came from.
2. Paragraphs should include a minimum of 4 sentences. The authors should group short paragraphs that present the same topic into one paragraph.
3. The background information about the effectiveness of co-designing apps is weak. Given that co-design an app is the main objective of this study, this evidence needs to be strengthen.
4. In the last paragraph, conclude with a comment about whether the study’s aim was achieved.

**Materials and Methods**

1. What were the participants’ exclusion criteria?
2. What was the guiding framework/theory used by the authors to collect, analyze, and interpret the study’s data to co-design the app?
3. When the authors state “A previous study database was randomized and 136 individuals who met the eligibility criteria were contacted…” Which study are the authors referring to? How were the individuals randomized?
4. Specify who recruited the participants of this study (those who made the phone calls), who conducted the pre-interviews in the participants’ home, who conducted the focus-groups and co-design activity? What are the qualifications of these researchers?
5. Can the authors include an introductory sentence with the stages of the focus group? Were these stages conducted in one focus group session or several sessions? It is not clear if the subsequent co-design activity in which the participants were sub-grouped into digitally engaged or not happened within the focus group or was separate activity.
6. Where were the focus group conducted?
7. The sentence of ethical approval should be moved to the first sentence
8. Data analysis:
   a. Add a section of data analysis at the end of the Materials and Methods section.
   b. What was the methods used for processing data prior to and during analysis, including transcription, data entry, data management and data coding?
a. State the authors or guidelines used by the authors to conduct the content analysis, not only the reference and the process by which inferences and themes were identified and developed, including the researchers involved in data analysis.
b. What were the techniques used to enhance trustworthiness and credibility of data analysis?
c. Consider changing “Simple statistical operations” for “Descriptive statistics”. Include all the descriptive statistics used to analyze the quantitative data concerning the participants’ characteristics (in addition to percentages and frequencies).
9. Procedures:
   a. Please, describe the “PAUL” project.
   b. Add references to the following methodological action: “This reduced the risks of induction through the researcher’s own posture and in the expression of the statements, as well as respect for the instructions for applying each instrument.”
a. How “Active and healthy aging, motivation for physical activities in urban areas and applications for monitoring and training” were addressed during the focus groups? They received information about these topics from the researchers or these topics were addressed through the focus group questions?
b. When the authors state “The participants gave their opinion, answered and discussed the issues before the group,” what does “before” mean? It is before the focus groups were conducted?
c. The authors need to add references, explain and justify the “Keep, Loose and Change method” for Re –design. Why was that method chosen among the others?
d. Authors should group short paragraphs that address the same topic.

**Results:**

1. An introductory paragraph should be included presenting the themes and sub-themes that emerged form the focus groups data.
2. The results section needs to be better organized. If content analysis was used to develop the categories, why a Table 2 was inserted halfway of the result’s section with only some of the categories?
3. The authors already stated in the methods section that a content analysis was used. No need to restate this in the results section.
4. It is not clear why the authors inserted the Table 2 at the middle of the results section. If the authors used thematic content analyses for the presenting the results
5. Specify how many participants were in each sub-group.

**Conclusions:**

1. I recommend to change the “Conclusion” subheading for “Discussion.”
2. Start this section by re-stating the purpose of your study.
3. Add references to the following statement: “Focus groups have traditionally been used in market and design research.
4. Highlight what is new about your study.
5. Study limitations are missing and must be established.

**General recommendations:**

1. The manuscript requires to be proofread and edited for the English language.

This is a good study with unique population that does not get enough attention in the literature. I wish you the best and thank you for the opportunity to review your work.
